# Physicochemical properties, biological activities, applications, and protective potential of the skeletal system of *Eucommia ulmoides* polysaccharides: a review

**DOI:** 10.3389/fphar.2025.1570095

**Published:** 2025-03-13

**Authors:** Xudong Liu, Yan Xing, Guijun Liu, Dapeng Bao, Zhaojiong Zhang, Haizheng Bi, Meng Wang

**Affiliations:** ^1^ Heilongjiang University of Chinese Medicine, Key Laboratory of Basic and Application Research of Beiyao Ministry of Education, Harbin, China; ^2^ Nursing Humanities Teaching and Research Office, Heilongjiang Nursing College, Harbin, China

**Keywords:** *Eucommia ulmoides* Oliv, polysaccharides, physicochemical properties, biological activities, modification

## Abstract

*Eucommia ulmoides* Oliv (*E. ulmoides*) is a widely distributed plant with economic value, nutritional value, edible value and even medicinal value. In recent years, *E. ulmoides* polysaccharides are considered to be one of the most important bioactive ingredients in *E. ulmoides*. Modern pharmacological studies show that the crude extract of *E. ulmoides* polysaccharides, their active monomer and ramifications have a wide range of pharmacological activities *in vitro* and *in vivo* experiments, which can be used to improve inflammation, regulate immunity, improve osteoporosis, and promote osseointegration, etc. Therefore, this review focuses on the induction and summary of the research at home and abroad in recent years, and summarizes the extraction and purification, modification methods, physicochemical properties, biological activities and potential mechanisms of *E. ulmoides* polysaccharides, providing a theoretical basis for the in-depth study of *E. ulmoides* polysaccharides and the development of related products.

## 1 Introduction

**FIGURE 1:**
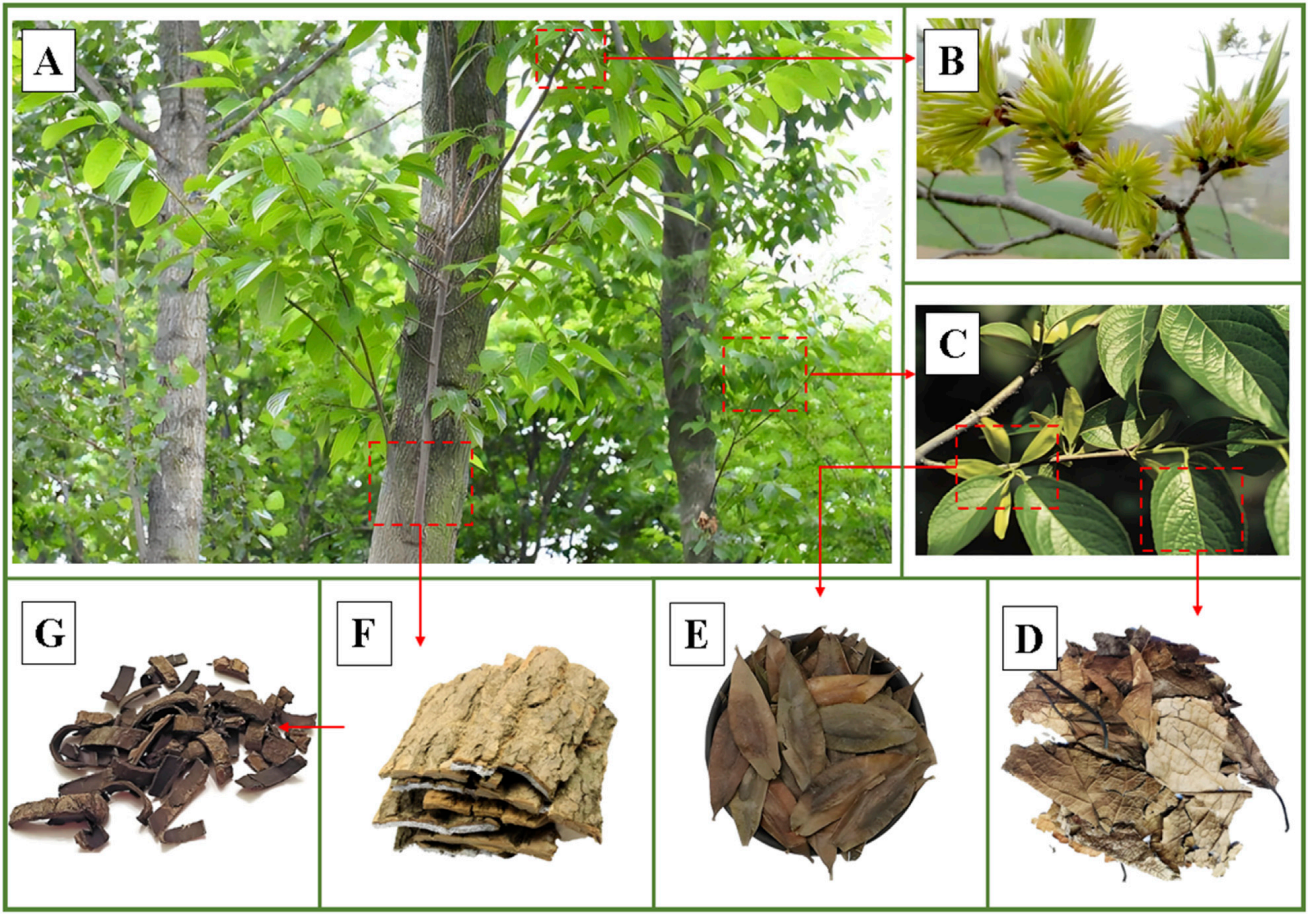
The plant morphology of *Eucommia ulmoides* Oliv. **(A)** Whole plant, **(B)** Flowers, **(C)** Leaves and fruits, **(D)** Dried leaves, **(E)** Dried fruits, **(F)** Barks, **(G)** Dried barks. (For intuitive introduction to the plant morphology of *Eucommia ulmoides* Oliv., the images are processed and quoted to show the characteristics of different parts from network and public sources).

*Eucommia ulmoides* Oliv (*E. ulmoides*) is a perennial deciduous tree belonging to the *Eucommia* genus (Yang et al., 2023). *E. ulmoides* is considered, on account of its economic, edible, nutritional and medicinal value, as “plant gold.” Its English name is “Eucommiae cortex,” which is also called “Du-Zhong” in China (Zhao et al., 2024). As an ancient species, it has existed for more than 2 million years, but the presence of the Quaternary glacial period has led to the fact that *E. ulmoides* only exists in subtropical and southern temperate zones (Xu et al., 2023). *E. ulmoides* thrives in warm, humid climates with ample sunlight, typically found in sparse forests at altitudes of 300–500 m (Liu et al., 2023). Nevertheless, this species is remarkably resilient, capable of enduring extreme cold and adapting to a wide range of soil types, which contributes to its widespread distribution across China. Among the various parts of *E. ulmoides*, its barks are the main medicinal ingredients, which have the effects of antioxidant, antihypertensive and weight loss, and are included in the Chinese Pharmacopoeia 2020 edition by Chinese Pharmacopoeia Commission (Yan et al., 2022). Notably, the leaves of *E. ulmoides* exhibit pharmacological effects equivalent to those of the barks, suitable for both medicinal and culinary purposes, as illustrated in Figure 1 (Yan et al., 2018). Its leaves have both edible value and medicinal value, which have the functions of antihypertensive and tonifying liver and kidney, and are listed in the list of “homology of medicine and food” by the National Health Commission of the People’s Republic of China (NHC, PPC) (Ren et al., 2023; Liang et al., 2024a). Simultaneously, it has gained significant interest from food scientists, nutritionists, pharmaceutical researchers, and health-focused consumers due to its rich content of nutrients, trace elements and bioactive ingredients.

The extensive history of *E. ulmoides* and research indicates its multiple values. It is an edible healthy food that provides multiple benefits in the daily diet and is also a natural medicinal plant providing a source of bioactive ingredients used in empirical medicine (Wu et al., 2018). In the realm of traditional Chinese medicine, every part of the *E. ulmoides* has values. Ingredients found in its flowers, fruits, leaves, bark, and other parts demonstrate beneficial bioactivities and offer health benefits (Ma et al., 2022). In particular, the primary active ingredient of *E. ulmoides* flowers is quercetin, which demonstrates exceptional hypoglycemic effect, and the inclusion of lignin and tannin further enhances its ability to stimulate gastrointestinal motility (Chen et al., 2023). *E. ulmoides* fruits are abundant in iridoid glycosides, showcasing robust anti-tumor effect (Liu et al., 2014). *E. ulmoides* leaves contain bioactive ingredients including chlorogenic acid and geniposide, offering significant benefits in protecting the cardiovascular system, and exhibiting antibacterial effects (Zhang et al., 2024a). *E. ulmoides* barks are rich in aucubin and polysaccharides, providing anti-inflammatory and immune modulation effects (Jia et al., 2019). Its edible, medicinal properties and rich nutritional value have been deeply explored and widely used. Its importance is in line with the growing demand of modern consumers for healthy diets, nutritional supplements and economic benefits.

Recent decades of research have demonstrated that phytochemicals and bioactive ingredients, including flavonoids, lignans, iridoids, and phenylpropanoids, exhibit a range of biological activities and offer health benefits (Guo et al., 2020). However, micromolecular ingredients typically lack the ability to specifically target, potentially affecting non-target tissues. Whereupon, research is increasingly shifting towards macromolecular polysaccharides. *E. ulmoides* is rich in bioactive polysaccharides of various types, exhibiting distinct and notable biological activities, including anti-osteoporosis, anti-inflammatory, immune modulation, promoting osseointegration, protection against alcoholic liver and brain injuries, among others (Xiao et al., 2023; Gong et al., 2022). Research indicates that *E. ulmoides* polysaccharides can enhance intestinal flora, restore intestinal mucosal permeability, and decrease endotoxins, inflammatory markers that may contribute to obesity, type 2 diabetes, fatty liver, and atherosclerosis. Consequently, *E. ulmoides* polysaccharides have the potential to regulate blood glucose, mitigate chronic inflammation, ameliorate atherosclerosis, and diminish obesity (Liang et al., 2024; Zhou et al., 2023). This positions them as a promising ingredient for health products or pharmaceuticals. At present, using various extraction and purification methods, a diverse array of polysaccharides has been extracted from different parts of *E. ulmoides*. Each type of polysaccharide possesses a unique chemical structures and distinct biological activities, presenting significant application potential and product development promise.

Nonetheless, the extraction process of *E. ulmoides* polysaccharides requires innovation. Structural characterization primarily focuses on the primary structures, and the specific mechanisms of their biological activities require further exploration. Therefore, this paper provides the comprehensive review of research progress on the extraction process, structural characteristics, biological activities, and structure-activity relationships of *E. ulmoides* polysaccharides. Various applications of *E. ulmoides* polysaccharides and the status of related patents are introduced in detail, which provides an important reference for further research of *E. ulmoides* polysaccharides. In addition, the existing research defects of *E. ulmoides* polysaccharides are analyzed to provide ideas for future research.

## 2 Extraction, purification, and modification of *E. ulmoides* polysaccharides

### 2.1 Extraction

Refined *E. ulmoides* polysaccharide was obtained through four steps of pretreatment, extraction, separation and purification (Chen and Yang, 2024; Qu et al., 2013). Before polysaccharides extraction, the *E. ulmoides* bark, leaves, *etc.* were typically washed and dried. They were then crushed, sieved through a 60-mesh sieve, and mixed with petroleum ether or 95% ethanol. The mixture was refluxed or sonicated to eliminate micromolecular ingredients like gums, lipids, cellulose, and pigments. Then different methods were used to extract solid residues, such as hot water extraction (HWE), conventional hot reflux extraction (CHE), ultrasonic assisted extraction (UAE), microwave-assisted extraction (MAE), *etc*. Owing to its simplicity and safety, HWE stands as the go-to method for polysaccharide extraction (Zhang et al., 2024b). Existing literature indicated that under the conditions of an extraction temperature between 80°C and 100°C, a duration of 80–180 min, 2-3 extractions, and a solid-liquid ratio of 1:3 or 1:20, the extraction yield of *E. ulmoides* polysaccharides using HWE ranged from 2.0% to 23.9%, as shown in Table 1. However, due to the lengthy duration of HWE, new extraction methods had emerged. UAE is a technique that employs ultrasound to intensively agitate *E. ulmoides* polysaccharide particles, leading to the rupture of cell walls and membranes to release the polysaccharides (Hromádková et al., 2002). This approach was primarily employed in extracting polysaccharides from *E. ulmoides* leaves. The extraction temperature was typically 60°C, with an extraction time ranging from 80 to 120 min, a power of 200 W, and a solid-liquid ratio of 1:20 or 1:30 (Feng et al., 2022; Feng et al., 2021; Liu et al., 2020). Under these conditions, the yield of *E. ulmoides* polysaccharides can peak at 16.495%, which was generally higher than that achieved by HWE, and the process was time-efficient (Liu et al., 2020). It is worth noting that higher ultrasonic power can enhance the disruption of *E. ulmoides* polysaccharides and accelerate the release rate of polysaccharides. However, excessively high power can degrade the *E. ulmoides* polysaccharides, and excessively long processing times can similarly lead to the degradation of polysaccharides. Therefore, in the process of ultrasonic extraction of *E. ulmoides* polysaccharides, it is necessary to pay attention to the selection of ultrasonic power and processing time. Furthermore, when the extraction temperature was 74°C, the extraction time was 15 min, and the solid-liquid ratio was 1:29, the yield of *E. ulmoides* polysaccharide EULP-MAE from the leaves using MAE was 12.310%, whereas the yield of polysaccharide EULP-CHE from the leaves using CHE was 5.620% (Xu et al., 2018). The superior extraction yield of EULP-MAE maybe attributed to the microwave treatment, which disrupted the cells of the *E. ulmoides* leaves, thereby enhancing the dissolution of polysaccharide ingredients in the solvent. These auxiliary extraction methods have improved efficiency, shortened processing time, reduced energy and solvent consumption, providing ideas for the extraction of polysaccharides from other plants. Nevertheless, the present extraction methods for *E. ulmoides* polysaccharides remain rather limited. Combining multiple methods may best fulfill practical requirements and foster deeper exploration into the biological properties of *E. ulmoides* polysaccharides.

**TABLE 1:** A summary of *E. ulmodies* polysaccharides extraction, purification.

Part	Extraction						Purification		Ref
	Total polysaccharide fraction	Extraction	Time (min)	Temperature (°C)/P (W)	Solid–liquid ratio	Total yield (%)	Polysaccharide fraction	Purification method	
*E. ulmoides* leaves	Crude polysaccharide	UAE	120 min	60°C	1:20	N/A	PsEUL	Sevag, Sephadex G-150, macroporous adsorption resin (D101), DEAE Sephadex™ A-25	Feng et al. (2021); Feng et al. (2022)
*E. ulmoides* leaves	EULP-MAE	MAE	15 min	74°C	1:29	12.310%	N/A	N/A	Xu et al. (2018)
	EULP-CHE	CHE	15 min	74°C	1:29	5.620%	N/A	N/A	
*E. ulmoides*	EUP	HWE	80 min × 3	100°C	1:3	23.900%	N/A	N/A	Hong et al. (2013)
*E. ulmoides* leaves	Crude polysaccharide	HWE	N/A×3	80°C	1:20	N/A	ELP	DEAE-52 cellulose column	Cui et al. (2023)
*E. ulmodies* cortex	cEuOCP	HWE	180 min × 2	100°C	1:20	N/A	EuOCP-a, EuOCP-b EuOCP-c EuOCP3	DEAE-52 anion exchange column, HiPrep™26/60™™S-400, Ezload 26/60 Chromdex 200 pg	Song et al. (2023)
*E. ulmodies* barks	Crude EUPS	HWE	N/A×3	N/A	N/A	N/A	EUPS	Sevag, macroporous adsorption resin (D101), DEAE Sephadex™ A-25	Feng et al. (2016)
*E. ulmodies* barks	Crude EUPs	HWE	120 min × 3	N/A	N/A	2.200%	EUPs	TCA	Jiang et al. (2011)
*E. ulmodies* barks	total EUP	HWE	180 min × 3	100°C	N/A	N/A	EUP1 EUP2 EUP3	Sevag, DEAE 52-cellulose, Sephadex G-100	Li et al. (2017)
*E. ulmoides* leaves	Cp	UAE	80 min	60°C/200 W	1:30	16.495%	Pp	Sevag, hydrogen peroxide method and activated carbon method, DEAE-52 anion-exchange chromatography column	Liu et al. (2020)
*E. ulmodies* barks	Crude polysaccharide	HWE	N/A	N/A	N/A	2.000%	EWDS-1	DEAE Sepharose™ Fast Flow column, Sephacryl™ S-400, TCA	Zhu et al. (2008)
*E. ulmoides.* Leaves	N/A	HWE	N/A×2	100°C	1:20	N/A	EUPS	1% papain	Li et al. (2024a); Li et al. (2024b)
Purchase	EUP (purity: 60%)	N/A	N/A	N/A	N/A	N/A	N/A	N/A	Sun et al. (2021)

N/A: means not mentioned.

### 2.2 Purification

**FIGURE 2:**
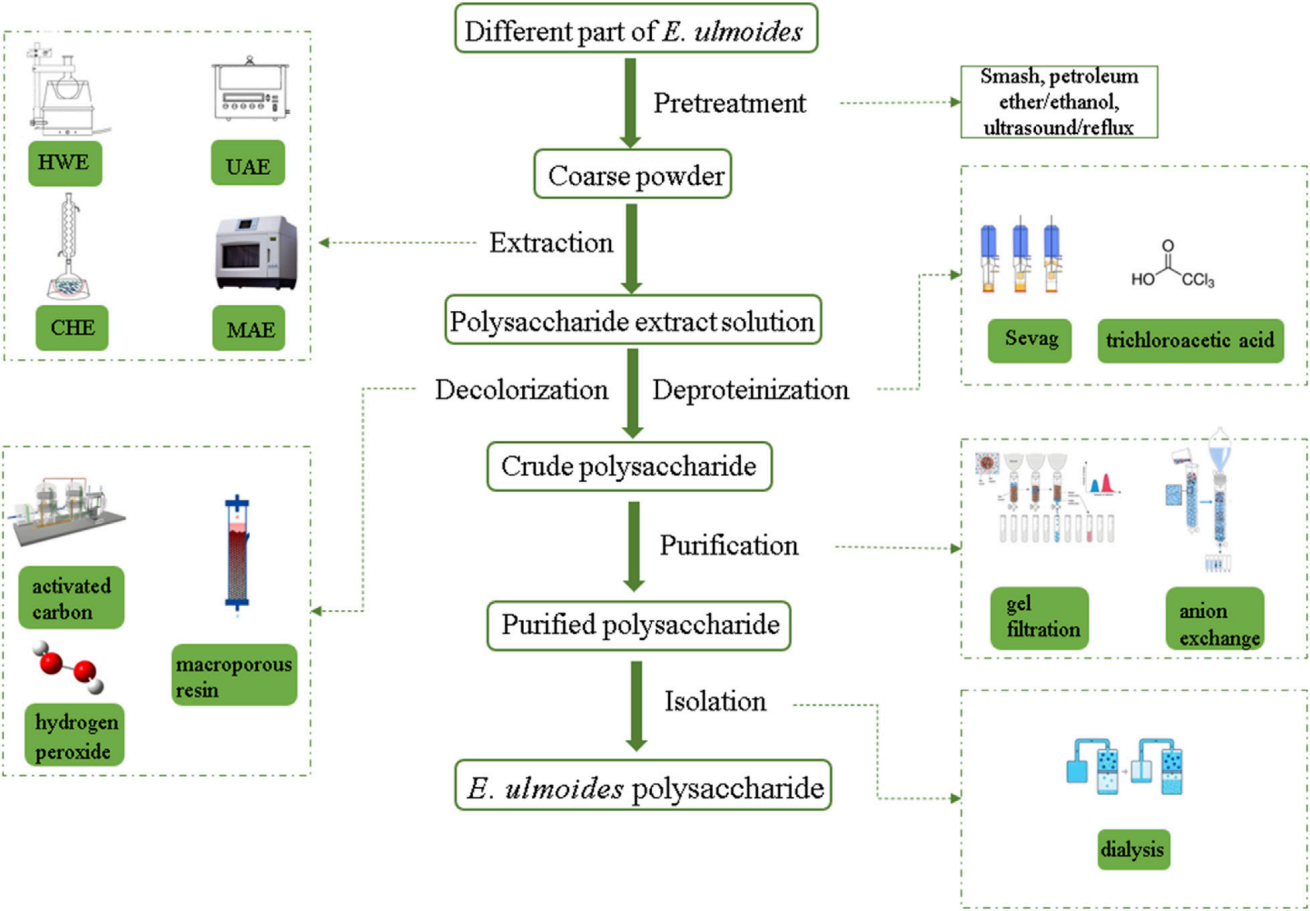
Flow chart of *E. ulmodies* polysaccharides extraction and purification.

Further purification of crude polysaccharides is required in order to obtain homogeneous polysaccharide fractions and to determine their structural characteristics (Borazjani et al., 2018). Traditionally, crude *E. ulmoides* polysaccharides were first deproteinized by treatment with Sevag and trichloroacetic acid, followed by decolorization with hydrogen peroxide, activated carbon or macroporous resin adsorption (Zhang et al., 2020). Then, with relevant absorbent and eluent as mobile phase, using column chromatography for further purification (Wang et al., 2020). Ion exchange chromatography and gel filtration chromatography are two common column chromatography, separation of they have different features. Generally, ion exchange chromatography can be used for the separation of neutral or acidic polysaccharides, and the commonly used chromatographic media for the separation of polysaccharides from *E. ulmoides* included diethylaminoethyl (DEAE) 52-cellulose and DEAE Sephadex A-25 (Michalski and Kończyk, 2024). For gel filtration chromatography, Sephadex G-100, Sephadex G-150 and Sephacryl S-400 were commonly used as the chromatographic media for the separation of different polysaccharides (Khan et al., 2024a). The purified *E. ulmoides* polysaccharide fraction was then obtained by concentration, dialysis and lyophilization. Finally, the *E. ulmoides* polysaccharides and protein contents of the purified products were determined by phenol-sulfuric acid method and Bradford method, respectively (Yu et al., 2018). It is worth noting the effects of different extraction parts and methods. Different extraction parts result in varying proportions of active ingredients, which in turn influence the frequency and duration of deproteinization for *E. ulmoides* polysaccharides. This, in turn, affects the content and yield of these polysaccharides. In addition, although organic reagents have not been used for the extraction of *E. ulmoides* polysaccharides, protein denaturation induced by organic reagents should be considered in future studies. The schematic diagram of the extraction and purification process of *E. ulmoides* polysaccharide is shown in Figure 2.

### 2.3 Modification

Naturally occurring macromolecules, particularly plant polysaccharides, possess unique biological structures that endow them with pronounced biological activities (Jia et al., 2024b). Introducing chemical groups to generate modified polysaccharides is crucial for conferring diverse structures, which impart useful properties to these polysaccharides (Li et al., 2024a). The modification information of *E. ulmoides* polysaccharides has been listed in detail in Table 2.

**TABLE 2:** A summary of *E. ulmodies* polysaccharides modification methods.

Part	Name of polysaccharide	Modification method	Ref
*E. ulmoides* leaves	PsEUL-OVA	PsEUL + OVA + EDC	Feng et al. (2021)
*E. ulmoides* leaves	PsEUL-OVA/Cubs	PsEUL-OVA + phytotriol + F127	Feng et al. (2022)
Purchase EUPS (purity ≥97%)	anti-DEC-205-EUPS-OVA-LPSM	OVA + EUPS + liposome + anti-DEC-205	Feng et al. (2020)
Purchase EUP (Haochen Biology)	EUP-Sr	EUP + Sr	Deng et al. (2019)
Purchase EUP (Haochen Biology)	Ti-EUP-Sr	EUP-Sr + Ti	Sun et al. (2023)
Purchase EUP (Haochen Biology)	DPEEK@EUP-Sr	PEEK + EUP + Sr	Zhang et al. (2022)
Purchase EUP (Tianrui Biology)	EUP-SeNP	EUP + SeNP	Ye et al. (2023)
Purchase EUPS (purity ≥96.5%)	EUPS-PLGA anti-DEC-205-EUPS-PLGA NPs	EUPS + PLGA DEC-205 + anti-CD205 MAb	Feng et al. (2023)

Strontium (Sr) is a trace element in the human body that can inhibit inflammation and plays an important role in the osteoclast genesis and osteoblast genesis systems of animals (Xiao et al., 2023). Mix a saturated aqueous solution of *E. ulmoides* polysaccharides EUP, sourced from Haochen Biology, with an equivalent volume of a 2M SrCl2 aqueous solution. Subsequently, NaOH was added to adjust the pH to 9–11, and the reaction lasted for 1 h. Afterward, anhydrous ethanol was introduced into the reaction mixture and left to stand for 24 h to ensure thorough precipitation of the composite powder. The resultant composite powder was filtered using a vacuum suction device, then the powder was stirred and washed with ethanol aqueous solutions of varying concentrations (80%, 60%, 40%) to obtained EUP-Sr (Deng et al., 2019). Recent studies suggest that EUP-Sr may inhibit the production of inflammatory factors and osteoclasts (OC), while enhancing osteoblast expression, positioning it as a promising biomaterial for bone regeneration immunotherapy (Deng et al., 2019).

Titanium (Ti) implants are a crucial element in tooth replacement, yet their insertion frequently leads to inflammation of the surrounding tissues (Addy, 2024; Kheder et al., 2023). Consequently, modifications and coatings on the implant surface are necessary to strengthen bone integration and alleviate inflammatory responses, with EUP-Sr emerging as a top choice in research (Liu et al., 2024a). The titanium sample undergoes hydrothermal alkaline treatment to create a uniform porous structure on its surface, followed by coating with a polydopamine layer to obtain Ti-PDA for surface modification. Subsequently, EUP-Sr is dissolved in a 10 mM Tris-HCl buffer to prepare a saturated solution of EUP-Sr. Then, Ti-PDA slices are immersed in this solution to yield Ti-EUP-Sr (Sun et al., 2023). Research has shown that introducing EUP-Sr on the surface of titanium can significantly enhance its anti-inflammatory, angiogenic, and osteogenic abilities, thereby achieving better bone integration effects (Sun et al., 2023). Additionally, EUP-Sr can bind with polyetheretherketone (PEEK) that has been surface-modified with dopamine (DPEEK), forming DPEEK@EUP-Sr. This improves the inert biological properties of PEEK and boosts its bone integration activity (Zhang et al., 2022).

Selenium (Se) is an essential trace element in the human body, with strong anti-inflammatory, antioxidant, and anticancer biological activities (Ansari et al., 2024). SeNP, a novel form of selenium, exhibits enhanced antioxidant activity, superior bioavailability, and reduced toxicity compared to Se (Nag et al., 2024). However, SeNP is highly unstable in liquid solutions, prone to reducing its bioavailability and bioactivities (Nwoko et al., 2021). Thus, appropriate stabilizers are essential to enhance its stability. Studies have demonstrated that biological macromolecules, such as polysaccharides, can modify SeNP, resulting in enhanced biological activities. Sodium selenite and ascorbic acid are mixed in a 1:1 ratio and stirred for 1 h, followed by the addition of EUP, obtained from Tianrui Biology, to yield EUP-SeNP (Ye et al., 2023). Research indicates that employing EUP as a surface modifier to decorate SeNP results in EUP-SeNP, endowed with stable anti-inflammatory and antioxidant properties (Ye et al., 2023).

Antigenic proteins modified by polysaccharides can bolster the immune cells’ recognition of their antigens while maintaining their specific response to natural antigens (Kushwaha et al., 2024). An antigen delivery system using PsEUL-assisted OVA (PsEUL-OVA) was synthesized by coupling PsEUL to OVA using the EDC method, which was shown to enhance antigen-specific immune response (Feng et al., 2021). However, the release rate and antigen presentation efficiency of PsEUL-OVA need to be enhanced. Consequently, the amphiphilic lipid substance, plant triol, was incorporated into the aqueous solution of F127 (dispersion system) to induce the spontaneous formation of cubic micelles. Following ultrasonication, these micelles were dispersed into individual lipid cubes. PsEUL-OVA was encapsulated within these cubes to produce PsEUL-OVA/Cubs (Feng et al., 2022). Additionally, nanoparticles targeting the DEC-205 receptor have been found to induce antigen-specific protective immune responses (Tabansky et al., 2018). When the delivery system simultaneously carries antigens and immunomodulators, it can optimize the anticipated therapeutic efficacy of drugs and elicit robust humoral and cellular immune responses to the antigens (Dhodapkar et al., 2014). Antigens and EUPS (with a purity of ≥97%) were incorporated into nanoliposomes (EUPS-OVA-LPSM) using the membrane dispersion technique, followed by conjugation with DEC-205 antibodies to yield anti-DEC-205-EUPS-OVA-LPSM (Feng et al., 2020). An alternative approach involved encapsulating EUPS (with a purity of ≥96.5%) into PLGA nanoparticles (EUPS-PLGA) using a double emulsion solvent evaporation method. By linking anti-CD-205 monoclonal antibodies to the EUPS-PLGA nanoparticles, DEC-205 receptor-targeted EUPS-PLGA nanoparticles (anti-DEC-205-EUPS-PLGA NPs) were successfully prepared (Feng et al., 2023). Experimental findings indicate that both anti-DEC-205-EUPS-OVA-LPSM and anti-DEC-205-EUPS-PLGA NPs, serving as targeted adjuvants, enhance the maturation of dendritic cells and stimulate both humoral and cellular immune responses (Feng et al., 2023).

## 3 Physicochemical properties of *E. ulmoides* polysaccharides

A plethora of *E. ulmoides* polysaccharides have been identified, with their structural characteristics and microscopic features being pivotal to their biological activities (Chen et al., 2024a; Nuerxiati et al., 2021). At present, the structural characteristics and microscopic features of the existing *E. ulmoides* polysaccharides have been analyzed by Fourier transform infrared spectroscopy (FT-IR), scanning electron microscope (SEM), laser particle size analysis, gel permeation chromatography (GPC), high performance anion exchange chromatography (HPAEC), high performance liquid chromatography (HPLC), nuclear magnetic resonance (NMR), gas chromatography (GC), gas chromatography-mass spectrometry (GC-MS), high performance gel permeation chromatography (HPGPC), high performance capillary electrophoresis (HPCE), ultraviolet spectroscopy (UV), X-ray diffraction (XRD), atomic force microscope (AFM), energy dispersive spectrometer (EDS), thermogravimetric analysis (TGA), differential scanning calorimetry (DSC), and x-ray photoelectron spectroscopy (XPS). The detailed structural information of *E*. *ulmoides* polysaccharides and their derivatives is shown in Table 3.

**TABLE 3:** Physicochemical properties of *E. ulmodies* polysaccharides.

Source	Compound name	Molecular weights	Monosaccharide composition	Structures	Microscopic features	Analytical techniques	Ref
*E. ulmoides* leaves	PsEUL	N/A	Glu: Ara: Gal: Rha = 38.2–39.1: 37.7: 12.8: 11.8	PsEUL: FT-IR: 3422cm^−1^ is O—H, 2922cm^−1^ is C—H, 1654cm−1 is C=O of pyranose ring, 866cm−1 is C—H of β-pyran PsEUL-OVA: FT-IR: 3422cm^−1^ is O—H, 1514cm−1 is NH, 1124cm−1 is CN, 1580cm−1 is C=O	SEM: 100x time: The PsEUL was a relatively smooth and dense mass with a large number of fine particles attached to the surface of the PsEUL-OVA Greater magnification: The surface of PsEUL-OVA was smoother than that of PsEUL Laser Particle-Size: average particle size (PsEUL-OVA) = 8.98 ± 2.39 nm, PDI (PsEUL-OVA) = 0.081 ± 0.01, average zeta potential (PsEUL-OVA) = 15.49 ± 0.3 mV	FT-IR, HPLC, SDS-PAGE, SEM, Laser Particle-Size	Feng et al. (2021)
	PsEUL-OVA	60–600 kDa	N/A				
*E. ulmoides* leaves	PsEUL-OVA/Cubs	N/A	N/A	N/A	TEM: It has hexahedron and cube structure Laser Particle-Size: average particle size (PsEUL-OVA/Cubs) = 381.68 ± 12.28 nm. PDI (PsEUL-OVA/Cubs) = 0.175 ± 0.01 nm, average zeta potential (PsEUL-OVA/Cubs) = −10.43 ± 0.3 mV.	TEM, Laser Particle-Size	Feng et al. (2022)
*E. ulmoides* leaves	EULP-MAE	3.8830 kDa	Rha: Ara: Gal: Glu: Xyl: Man: GluA: GalA = 7: 4: 6: 14: 1: 2: 3: 1	FT-IR: 3369cm−1 is -OH, 2926cm−1 is C—H, 1745cm−1 is C=O, 1652cm−1 is bound water, 1400-1200cm−1 is -COOH, 1200-1000cm−1 is C—O—C and C—OH, 1042cm-1 is C—C, C—O and C—O—C, 913cm−1 is β-pyranose, 892cm−1 is β-glucoside bond, 760cm−1 is β-merging.	N/A	GPC, HPAEC, FT-IR	Xu et al. (2018)
	EULP-CHE	1.2055 kDa	Rha: Ara: Gal: Glu: Xyl: Man: GluA: GalA = 9:32:20:55:19:2:14:1				
*E. ulmoides* leaves	ELP	N/A	Ara: GalA: Gal: Rha: Glu = 26.61: 25.1: 19.35: 16.13: 12.90	N/A	N/A	HPLC	Cui et al. (2023)
*E. ulmodies* barks	EuOCP3	38.1 kDa	Ara: GalA: Rha: Gal: Glu: GluA: Man: Fuc	FT-IR: 3361cm^−1^ is O—H, 2,923 and 1033cm−1 is C—H and C—O, 1733 and 1606cm−1 is uronic acid, 895cm−1 is α-glucosidic bond 1H and 13C NMR: δH 5.03, 5.19, 5.09, 5.06, 5.18, 5.12, 4.63 and 4.94 have eight different poly carbon signals in the δC 107.54, 107.15, 98.42, 101.44, 107.89, 97.48, 103.43 and 97.43, corresponding to H1/C1 of A-H, respectively. 1H-1HCOSY: Residue A: δH/H 5.03/4.07, 4.07/4.15, 4.15/4.35, and 4.35/4.71. Residue B: δH/H 5.19/4.10, 4.10/3.94, 3.94/4.15, and 4.15/3.66. Residue C: δH/H 5.09/4.05, 4.05/3.89, 3.89/3.49, 3.49/3.74, and 3.74/1.19. HSQC: Residue A:δH/C 5.03/107.54, 4.07/70.22, 4.15/76.58, 4.35/80.84,and 4.71/71.26. Residue B: δH/C 5.19/107.15, 4.10/81.29, 3.94/77.36, 4.15/82.36, 3.66/66.81. Residue C: δH/C 5.09/98.42, 4.05/79.13, 3.89/67.89, 3.49/75.11, 3.74/71.33, 1.19/16.61. 1H-1H NOESY combining HMBC: Residue A: -A-A-, -A-B-, -A-C- and -A-E-. Residue B: -B-B-, -B-C-, -B-A-, and -B-E-. Residue C: -C-C-, -C-B-, -C-A- and -C-E-. Residue D: -D-B-, -D-A- and -D-E-. EuOCP3 was predominantly composed of →4)-α-GalpA-(1 → 4)-α-GalpA-(1→, →4)-α-GalpA-(1 → 5)-α-Araf-(1→, →4)-α-GalpA-(1 → 2)-α-Rhap-(1→, and →4)-α-GalpA-(1 → 5)-α-Araf-(1 → 2)-α-Rhap- (1 → repeating blocks, which were connected by →2,3,5)-α-Araf-(1→. The side chains, substituted at C-2 and C-5 of →2,3,5)-α-Araf-(1→, contained T-β-Araf→ and T-β-Araf → 4)-α-GalpA-(1→ residues.	N/A	1-phenyl-3-methyl-5-pyrazolone pre-column derivatization method, HPLC, GPC, NMR, FT-IR,	Song et al. (2023)
*E. ulmodies* barks	EUPS	1,146.32 kDa	Glu: Fru: Man: Fuc: Gal: Ara = 36.6: 16.6: 14.2: 15.7: 9.5: 7.4	FT-IR: 3447.35 cm−1 is O—H, 2,939.14 cm−1 is C—H, 1,637.37 cm−1 is C—O, 1,405.88 cm−1 is COO-,1,000–1200cm−1 is C—O—C and C—OH, 1,118.54 cm−1、1,153.24 cm−1 and1263.77 cm−1 are pyranose, 890.79 cm−1 is β-glycosidic bond.	SEM: EUPS is spongy with rough surface, pores and cracks	GPC, GC, GC-MS,SEM	Feng et al. (2016)
*E. ulmodies* barks	EUPs	N/A	Ara: Gal: Glu: Rha = 6.35: 3.15: 1.47: 1	N/A	N/A	GC, HPGPC	Jiang et al. (2011)
*E. ulmodies* barks	EUP1	358.1 KDa.	Rha: Ara: Gal: Man: Glu = 4.6: 8.6: 6.1: 1: 2	NMR: 1H NMR: major abnormal proton signals of δ5.22, 5.19, 5.07, 5.01, 4.98, 4.94, 4.92, 4.48 and 4.47 ppm 13C NMR: major abnormal proton signals of δ109.1, 107.4, 107.3, 107.0, 104.2, 103.2, 101.3 and 98.3 ppm δ78.9, 79.1, 80.7, 81.1, 82.1, 82.2 and 83.7 ppm are C-2, C-3 or C-4. δ66.7, 66.3, 66.1, 62.7, 60.9, 60.8 and 60.6 ppm are C6(C5). The EUP1 linkage of →3,4-rha–1 → 3-glc-1→, →4-Man–1 → 4-Glc-1→, →4-Glc–1 → 4-Glc-1→, →4-Glc–1 → 3-Glc-1→, →3-Glc–1 → 4, 3-Rha–1→, →3,4-Rha–1 → 3,6-Gal-1→, →3,6-Gal–1 → 3,6-Gal–1 → 6-Gal-1→, →6-Gal–1 → 6-Gal–1 → 3-Gal-1→, Man–1 → 3,6-Gal-1→.	N/A	GC–MS, FT-IR, NMR, HPLC	Li et al. (2017)
*E. ulmoides* leaves	Pp	N/A	N/A	FT-IR: 3417cm−1 is O—H, 2930cm−1 and 1406cm−1 are C—H, 1591cm−1 is C=O, Strong banding between 950cm and 1 and 1160cm−1 is pyranose ring. 866cm−1 is α-ectopic configuration. Strong banding between 2142cm and 1 and 2050cm−1 is protein peak.	N/A	FT-IR	Liu et al. (2020)
*E. ulmodies* barks	EWDS-1	2000 kDa	Gal: Glu: Ara = 2.1: 1.0: 0.9	FT-IR: 3446cm−1 is O—H, 2921cm−1 is C—H, GC-MS: there are terminal, 1,5-linked and 1,3,5-linked α-L-Araf, terminal, 1,4-linked, 1,6-linked, and 1,4,6-linked β-D-Galp, terminal, 1,3-linked and 1,4-linked β-D-Glcp. The identification of 1,3,4,5-tetra-O-acetyl-2-O-methyl-arabinose (1,3,5-linked α-L-Araf), 1,4,5,6-tetra-O-acetyl-2-O-methyl-galactose (1,4,6-linked β-D-Galp) NMR: δ109.2 and δ107.6 are α-L-Araf, δ104.1-δ105.2 are β-D-Galp and β-D-Glcp.	N/A	NMR, HPCE, HPGPC, GC-MS, FT-IR	Zhu et al. (2008)
*E. ulmoides* leaves	EUPS	186.132 kDa	Fuc: Rha: Ara: Gal: Glu: Xyl GalA: GluA: Man = 0.48: 4.42: 18.69: 29.46: 35.9: 3.19: 3.82: 2.52: 1.5	N/A	N/A	GPC, HPAEC	Li et al. (2024a); Li et al. (2024b)
Purchase	anti-DEC-205-EUPS-OVA-LPSM	N/A	N/A	N/A	It is a double membrane spherical or nearly spherical structure. anti-DEC-205-EUPS-OVA-LPSM showed an average particle size of about 200 nm	TEM, Laser particle size analyzer	Feng et al. (2020)
	EUP	3.170 kDa	GulA: Rib: Rha: Glu: GluA: Gal: Xyl = 0.81: 77.7: 4.44: 2.07: 1.15: 7.76: 6.06	UV: EUP has a peak at 280 nm, and EPU-Sr has no peak at 280 nm. FT-IR: 3424cm−1 is O—H, 2925cm−1 is C—H, 1,612 and 1415cm−1 are O—C—O, 1100-1000cm−1 is C—O—C and C—OH, 1042cm−1 is C—O and C—C (EUP), 1,000 cm−1 is C—O and C—C (EUP-Sr) NMR: α-heteropolymer protons occur at δ3.0-5.5ppm Indicates the presence of α-galactose (δ5.29 ppm).δ3.4-4.4 ppm is a ring proton. The 1.31 ppm and 1.24 ppm signals come from L-rhamnose, it is allocated to O-2- and O-2, 4-connected rhamnoses, respectively.	SEM: compared with EUP, EUP-Sr has smaller particle size and more uniform distribution. EDS: 26% of the Sr was in the EUP-Sr XRD: EUP is a semi-crystalline substance. AFM: both EUP and EUP-SR samples were spherical with narrow height distribution. Average height of EUP is about 15 nm, average height of EUP-Sr is about 11 nm.	HPLC, GPC, UV, FT-IR, XRD, SEM, AFM, TGA, DSC, EDS	Deng et al. (2019)
	EUP-Sr	14.700 kDa	N/A				
	Ti-EUP-Sr	N/A	N/A	FT-IR: 3360cm−1is -OH, 2929cm−1 is C—H, 1100cm−1 is C—O—C and C—OH, 1619cm−1 is N—H, 1490cm−1 is C=C, 1281cm−1 is C—O and/or C—N, 1089cm−1→1,040 cm−1 is C—O.	SEM: homogeneous aggregates of EUP-Sr particles were observed on the surface of Ti-EUP-Sr. EDS: Strontium content (Ti-EUP-Sr-12H) = 6.94%, (Ti-EUP-Sr-24H) = 9.38%	SEM, EDS, FTIR,	Sun et al. (2023)
	EUP-SeNP	N/A	N/A	FT-IR: 3397cm−1 is O—H, 1383cm−1 is C—O—C, 2926cm−1 is C—H.	TEM: a monodisperse and uniform spherical structure.	TEM, FT-IR,	Ye et al. (2023)
	EUPS-PLGA	N/A	N/A	N/A	average particle size = 302.3 ± 3.19 nm PDI = 0.138 ± 0.041, Zeta = −4.2 ± 0.1 mV	Laser Particle-Size	Feng et al. (2023)
	anti-DEC-205-EUPS-PLGA NPs	N/A	N/A	N/A	average particle size = 321.77.3 ± 5.14 nm PDI = 0.138 ± 0.041, Zeta = −7.2 ± 0.1 mV		
	DPEEK@EUP-Sr	N/A	N/A	FT-IR: 1,653 cm−1 and 1,597.5 cm−1 are C=O, 1,124, 1,188.5 and 1,160 cm−1 are C—O—C.	SEM: The surface is a three-dimensional porous network structure with an aperture of about 1–3 μm	SEM, FT-IR, NMR, XPS	Zhang et al. (2022)

N/A: means not mentioned.

### 3.1 Molecular weight

The molecular weight (Mw) distribution of polysaccharides reflects the size range and relative content of molecules in polysaccharide samples, which has important guiding significance for studying the physical properties, structural functions, and relationship with biological activity of polysaccharides (Chen et al., 2024). *E. ulmoides* polysaccharides are typically analyzed for Mw using HPLC, GPC, HPGPC, and HPCE. Previous studies have reported a Mw range of 1.2055–2000 kDa for *E. ulmoides* polysaccharides. Thereinto, the Mw of *E. ulmoides* leaves polysaccharides ranges from 1.2055 to 600 kDa, while that of *E. ulmoides* barks polysaccharides ranges from 38.1 to 2000 kDa. The Mw of EUP and EUP-Sr, obtained commercially, are 3.170 kDa and 14.700 kDa, respectively, suggesting they are polysaccharides from *E. ulmoides* leaves (Deng et al., 2019). Notably, the Mw of polysaccharides from *E. ulmoides* leaves obtained via MAE (EULP-MAE) exceeds that from CHE (EULP-CHE), likely due to the enhanced disruption of cell walls by microwave treatment, thus facilitating the release of polysaccharides (Xu et al., 2018). These findings demonstrate that the extraction parts and methods can influence the Mw distribution of *E. ulmoides* polysaccharides. In general, the higher the Mw of plant polysaccharides, the lower the proportion of low Mw polysaccharides in their hydrolysis products, which may lead to higher bioavailability (Wasser, 2002). Therefore, to enhance their bioavailability, further optimization of the extraction and purification conditions for *E*. *ulmoides* polysaccharides is essential.

### 3.2 Monosaccharide composition

The monosaccharide composition can influence the chain structure and higher-order structure of polysaccharides, with the latter being a key determinant of polysaccharides activities (Hu et al., 2021; Reiser et al., 1992). Therefore, analyzing the monosaccharide composition of polysaccharides holds significant importance. The primary methods for analyzing the monosaccharide composition of *E*. *ulmoides* polysaccharides encompass the 1-phenyl-3-methyl-5-pyrazolone pre-column derivatization technique, along with GC, GC-MS, HPLC, and HPAEC. *E*. *ulmoides* polysaccharides are a type of heteropolysaccharide, typically composed of rhamnose (Rha), arabinose (Ara), mannose (Man), galactose (Gal), xylose (Xyl), glucuronic acid (GluA), glucuronic (Glu), galactose acid (GalA), and fucoidan (Fuc), each with varying molar fractions. The monosaccharide composition of polysaccharides from *E*. *ulmoides* leaves typically includes Glu, Ara, Gal, Rha, GluA, GalA, Man, Fuc, and Xyl, while the monosaccharide composition of polysaccharides from *E*. *ulmoides* barks is typically Glu, Ara, Gal, Rha, GluA, GalA, Man, and Fuc. Notably, Fructose (Fru) is only present in the polysaccharides from Eucommia ulmoides bark (EUPS) (Feng et al., 2016). Additionally, Ribose (Rib) is only present in the *E*. *ulmoides* polysaccharides from Haochen Biology (EUP _(Haochen Biology)_), with a composition of GulA: Rib: Rha: Glu: GluA: Gal: Xyl = 0.81: 77.7: 4.44: 2.07: 1.15: 7.76: 6.06, suggesting that EUP _(Haochen Biology)_ likely originates from polysaccharides derived from *E*. *ulmoides* leaves (Deng et al., 2019). The differences in monosaccharide composition and molar ratios of these *E*. *ulmoides* polysaccharides can be attributed to differences in extraction parts, separation and purification techniques, and analytical methods.

### 3.3 Chemical structure

Recent scientific research has reportedly produced a variety of natural *E*. *ulmoides* polysaccharides and their derivatives. However, due to constraints in identification methods and technologies, there remains a scarcity of published literature on the chemical structures of *E*. *ulmoides* polysaccharides. Nonetheless, the available structural data for *E*. *ulmoides* polysaccharides and their derivatives are summarized as follows.

**FIGURE 3:**
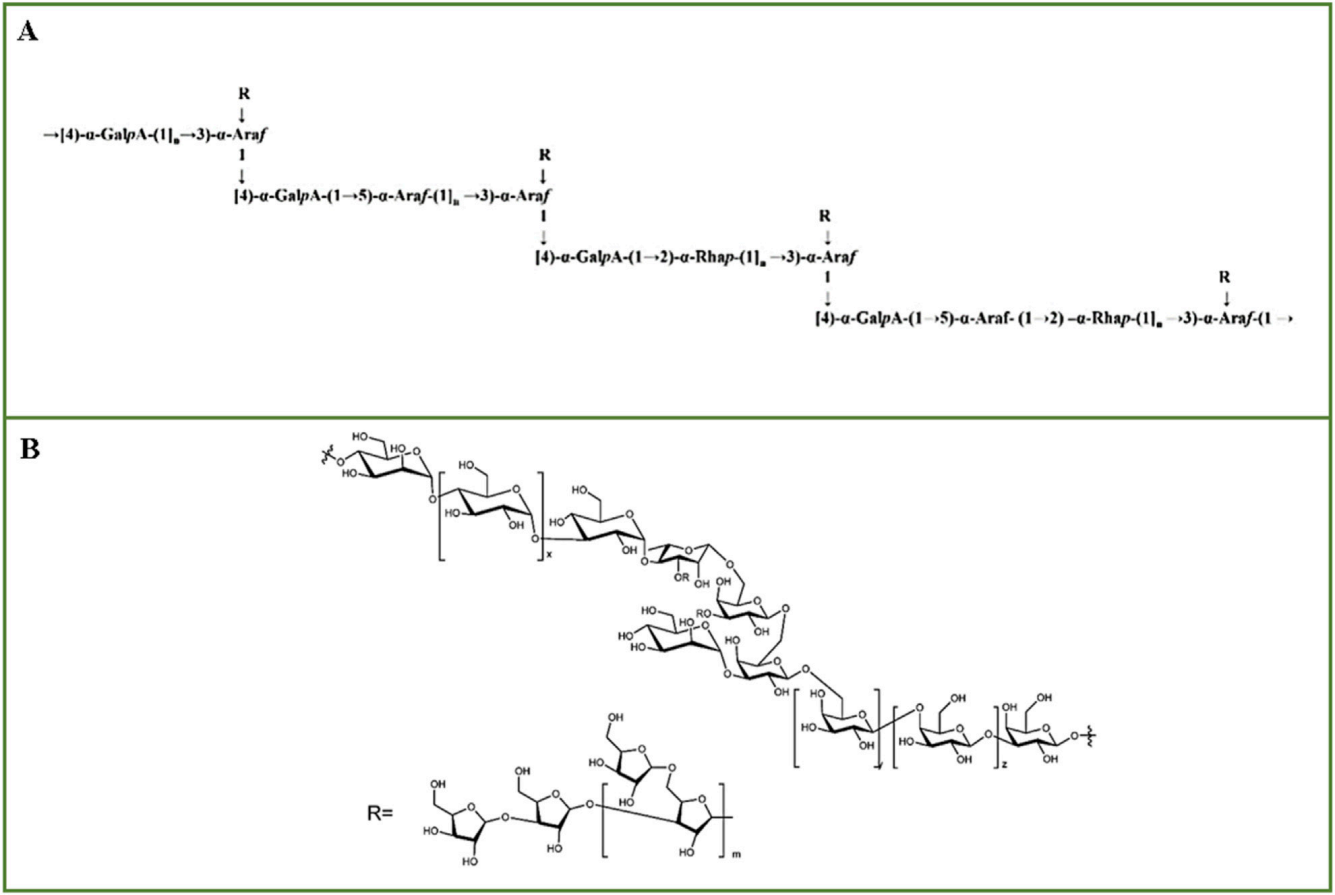
The possible structures. **(A)** The possible structure of EuOCP3 (Image from the literature Song et al., 2023), **(B)** The possible structure of EUP1 (Image from the literature Li et al., 2017).

Crude *E. ulmoides* cortical polysaccharide (cEuOCP) was purified sequentially by DEAE-52 anion exchange column, HiPrep™26/60™™S-400, Ezload 26/60 Chromdex 200 pg to obtain fraction EuOCP3 (Song et al., 2023). After methylation analysis, EuOCP3 was determined to be primarily composed of eight glycosidic residues, namely, T-Ara (f), 2-Rha (ha (p), T-Gal (p), 5-Ara (f), 2, 4-Rha (p), 4-Gal (p) (p) - UA, 4-Gal (p), and 2, 3, 5-Ara (f). Combining FT-IR and NMR, it is speculated that the backbone of EuOCP3 is mainly composed of → 4)-α- GalpA-(1 → 4)-α-GalpA-(1→, →4)-α-GalpA-(1 → 5)-α-Araf-(1→, →4)-α-GalpA-(1 → 2)-α-Rhap-(1→, and →4)-α-GalpA-(1 → 5)-α-Araf-(1 → 2)-α-Rhap- (1 → repeating blocks, consisting of → 2,3,5)-α-Araf-(1 → is the connection. The side chains are substituted at C-2 and C-5 of → 2,3,5)-α-Araf-(1 →, including T - β-Araf → and T - β-Araf → 4)-α-GalpA-(1 → residues, the possible structure of EuOCP3 is shown in Figure 3A (Song et al., 2023). The crude polysaccharide extracted from *E*. *ulmoides* barks was purified by DEAE 52 cellulose ion exchange column and Sephadex G100 gel filtration to obtain EUP1 (Li et al., 2017). Partial acid hydrolysis was conducted with various concentrations of TFA, and the hydrolysis of the main and branch chains of polysaccharides into monosaccharides was analyzed via HPGPC. GPC showed that acidic hydrolysis removed Ara from the backbone, inferring that Rha, Gal, Man, and Glu form the backbone of EUP1, while Ara is most likely in the branched chain. Through a combination of FT-IR, HPLC, NMR, and GC-MS analyses, the linkage of EUP1 was determined to be →3,4-Rha-1 → 3-Glc-1 →, →4-Man–1 → 4-Glu-1 →, →4-Glu-1 → 4-Glu-1 →, →4-Glu–1 → 3-Glu-1 →, →3-Glu–1 → 4, 3-Rha-1→, →3,4-Rha-1 → 3,6-Gal-1 →, →3,6-Gal–1 → 3,6-Gal-1 → 6-Gal-1 →, →6-Gal-1 → 6-Gal-1 → 3-Gal-1 →, Man-1 → 3,6-Gal-1 →. The possible structure of EUP1 is shown in Figure 3B (Li et al., 2017). The crude polysaccharide fraction extracted from the stem barks of *E*. *ulmoides* was further separated and purified by DEAE Sepharose™ Fast Flow column, Sephacryl™ S-400 to obtain EWDS-1 (Zhu et al., 2008). Methylation analysis showed that there were terminals, 1,5-linked and 1,3,5-linked α-L-Araf, 1,4-linked, 1,6-linked and 1,4,6-linked β-D-Galp, terminals, 1,3-linked and 1,4-linked β - D-Glcp. The identification of 1,3,4,5-tetraO-acetyl-2-O-methylarabinose (1,3,5-linked α-L-Araf) and 1,4,5,6-tetraO-acetyl-2-O-methylgalactose (1,4,6-linked β-D-Galp) indicates that EWDS-1 was a branched polysaccharide (Zhu et al., 2008).

Besides polysaccharides derived from the barks of *E*. *ulmoides*, those from its leaves have also been documented in research. PsEUL was an acidic polysaccharide originating from the leaves of *E*. *ulmoides*, inferred to consist of pyranose and furan rings based on FT-IR characteristics (Feng et al., 2021). Comparing the infrared spectra of PsEUL-OVA and PsEUL, it was determined that PsEUL effectively conjugated with OVA through the formation of covalent bonds (-CO-NH-) (Feng et al., 2021). The FT-IR spectrum of EULP-MAE reveals that microwave irradiation degraded the acetyl groups attached to the polysaccharide, resulting in only partial retention of the acetyl groups in EULP-MAE. Analysis of the FTIR spectra of EULP-CHE and EULP-MAE suggested that these polymers are β-type acidic heteropolysaccharides, featuring glucan groups and a high degree of branching (Xu et al., 2018). The FT-IR spectra of EUP-Sr and Ti-EUP-Sr revealed that the A peak in EUP-Sr at 1089 cm^−1^ was shifted to 1040 cm^−1^ in Ti-EUP-Sr (Sun et al., 2023). This shift can be explained by the formation of hydrogen bonds between the glycosidic bonds of EUP-Sr and the cyclic structure of PDA, supporting the hypothesis that PDA polysaccharide complexes can interact via intermolecular forces (Sun et al., 2023). The glycosidic bonds between polysaccharide moieties are crucial in determining the local conformation of polysaccharide chains, influencing their spatial structure, and the varied structures of *E*. *ulmoides* polysaccharides result in distinct orientations of their biological activities (Quintero et al., 2024). Consequently, the type of glycosidic bond is crucial for the diverse biological activities of *E*. *ulmoides* polysaccharides.

### 3.4 Microscopic features

Understanding the advanced structures of polysaccharides is crucial for elucidating their mechanisms of action (Liu et al., 2024b). By studying *E*. *ulmoides* polysaccharides through XRD, AFM, TEM, SEM, TGA, DSC, etc., the molecular and crystal structures of *E*. *ulmoides* polysaccharides can be understood, providing a theoretical basis for their pharmacological effects and applications.

SEM analysis revealed that under ×100 magnification, PsEUL appeared as a relatively smooth and dense aggregate, whereas the surface of PsEUL-OVA was adorned with a multitude of fine particles. At higher magnifications, the surface of PsEUL-OVA appeared smoother than that of PsEUL. Moreover, the average particle size of PsEUL-OVA was 8.98 ± 2.39 nm, with a polymer dispersity index (PDI) of 0.081 ± 0.01 nm and an average zeta potential of 15.49 ± 0.3 mV, signifying its commendable dispersibility, stability, and bioavailability (Feng et al., 2021). The TEM image of PsEUL-OVA/Cubs revealed its hexahedral and cubic structures, confirming successful encapsulation. Furthermore, with an average particle size of 381.68 ± 12.28 nm, PDI of 0.175 ± 0.01 nm, and an average zeta potential of −10.43 ± 0.3 mV, the material demonstrated excellent stability and dispersibility, and its sustained-release effect was superior to that of PsEUL-OVA (Feng et al., 2022). SEM analysis showed that compared with EUP, the particle size of EUP-Sr was smaller and more evenly distributed, which may be due to the favorable effect of strontium doping on optimizing the disordered structure of EUP. AFM analysis indicated that both EUP and EUP-Sr are spherical with a narrow height distribution. The average height of EUP was approximately 15 nm, whereas that of EUP-Sr was about 11 nm, possibly due to Sr introduction-induced aggregation of EUP molecular chains. XRD indicated that EUP is a semi crystalline ingredient, and the crystallinity of EUP-Sr generated after strontium doping had changed. TGA and DSC results showed that EUP-SR adsorbed more water and lost less total weight than EUP. This is because the conformation of the unstable structure may be “frozen” to form a steady state during the complex formation (Deng et al., 2019). It is worth noting that the average particle size of anti-DEC-205-EUPS-PLGA NPs was 321.77 ± 5.14 nm, but the particle size observed through TEM analysis was smaller. This discrepancy may result from the fact that the anti-DEC-205-EUPS-PLGA NPs were dried prior to detection, leading to the loss of their hydration film, and consequently, the particle size observed in a liquid environment showed greater variability (Feng et al., 2023).

## 4 Biological activities of *E. ulmoides* polysaccharides

### 4.1 Anti-inflammation activity

Inflammation is a set of protective responses elicited by stimuli within the body (Zhuang et al., 2022). Based on the etiology, inflammation is classified into infectious and aseptic types, and further divided into acute and chronic forms based on its duration (Helmberger and Kammer, 2005). Typically, acute inflammation subsides following the resolution of the infection, whereas chronic inflammation persists over time and can lead to organ dysfunction, cardiovascular diseases, metabolic syndrome, immune system disorders, and heightened cancer risk, thereby posing multiple health risks (Kotsifa et al., 2024; Pan and Ma, 2024). Therefore, the development of anti-inflammatory drugs is essential. Studies have found that bioactive ingredients, notably *E*. *ulmoides* polysaccharides, exhibit significant anti-inflammatory activities.

EUP1 is a polysaccharide obtained from *E*. *ulmoides* barks through HWE by Li et al. (2017). Cultured Raw 264.7 macrophages were treated with EUP1, and their phenotypic changes were assessed to ascertain their potential anti-inflammatory activities, as shown in Table 4. Results indicated that EUP1 stimulated the expression of CD206 and interleukin-10 (IL-10) in Raw 264.7 cells, without elevating the levels of the other two M2 markers, fizz1 or arg1. These findings suggest that EUP1 may exert anti-inflammatory activities on macrophages without inducing cells to adopt either polarization state. Then, *in vitro* and *in vivo* inflammatory models were constructed to evaluate the anti-inflammatory activity of EUP1. *In vitro* experiments, the levels of tumor necrosis factor (TNF-α) and IL-10 in the supernatant were measured using ELISA after treatment with different concentrations of EUP1 (10, 25, and 50 μg mL^−1^). The results indicated that EUP1 at concentrations of 10, 25, and 50 μg mL^−1^ significantly reduced TNF-α expression by approximately 25%, 40%, and 44%, respectively. *In vivo* experiments, a mouse sepsis model was established using lipopolysaccharide (LPS). Specifically, Male C57BL/6 mice were divided into three groups: Group I (saline), Group II (LPS at 10 mg·kg^−1^), and Group III (EUP1 at 10 mg·kg^−1^). The levels of TNF-α and interleukin-6 (IL-6) in the serum were detected using ELISA, and total RNA was extracted from the liver tissue to detect the RNA levels of TNF-α, IL-6, interleukin-1β (IL-1β), and IL-10. The results showed that EUP1 could effectively inhibit the expression of major inflammatory cytokines in septic mice, alleviate lung injury, and improve the survival rate of animals. In summary, EUP1 exhibited clear anti-inflammatory activity, with potential for further development as an anti-inflammatory drug (Li et al., 2017). Based on interactions involving glucan with toll-like receptor 2/DC-associated C-type lectin-1 (TLR-2/Dectin-1), glucan with recombinant mineralocorticoid receptor (MR), and galactose containing MGL presented saccharides, it is hypothesized that EUP1, can activate macrophages by binding to these carbohydrate receptors (Elieh Ali Komi et al., 2018). Activation of these receptors could initiate the expression of a blend of pro- and anti-inflammatory cytokines, with the latter potentially being significantly produced in the context of EUP1. Alternatively, polysaccharide EUP1, acting as an antagonist for LPS receptors like toll-like receptor 4/cluster of differentiation 14 (TLR4/CD14) or complement receptor 3 (CR3), could be involved in this process.

**TABLE 4:** Biological activities of *E. ulmodies* polysaccharides.

Biological activity	Polysaccharide name	*In vitro* or *in vivo*	Indicated concentration	Models/test system	Action or mechanism	Ref
Antioxidant activity	EUP	*in vivo*	300, 600 mg·kg^−1^	Healthy adult rabbits	EUP (300 mg·kg^−1^): MDA = 6.71 ± 0.54 GSH = 143.29 ± 11.09 EUP (600 mg·kg^−1^) MDA = 4.91 ± 0.35 GSH = 168.03 ± 12.49 SOD↑, CAT↑, GSH-Px↑, GR↑.	Hong et al. (2013)
	EULP-MAE	*In vitro*	0.1–2.0 mg·mL^−1^	DPPH	0.1–2.0 mg·mL^−1^ Superoxide anion radical clearance EULP-MAE > EULP-CHE. 2.0 mg·mL^−1^ (DPPH)IC_50_ (EULP-MAE) = 0.87 mg·mL^−1^ 2.0 mg·mL^−1^ (DPPH)IC_50_ (EULP-CHE) = 1.22 mg·mL^−1^	Xu et al. (2018)
	EULP-CHE	*In vitro*	0.1–2.0 mg·mL^−1^			
	EUP-SeNP	*In vitro*	N/A	DPPH,·OH, ABTS	The scavenging rates of DPPH,·OH and ABTS were increased by 59.41%, 79.30% and 68.11%, respectively。	Ye et al. (2023)
	Cp	*In vitro*	0.04–0.18 mg·mL^−1^	metal ion, DPPH, OH, ABTS	Cp: IC_50_ (metal ion) = 1.091 mg·mL^−1^, IC_50_ (DPPH) = 0.005 mg·mL^−1^, IC_50_ (OH) = 0.072 mg·mL^−1^, IC_50_ (OH) = 0.094 mg·mL^−1^ Pp: IC_50_ (metal ion) = 1.041 mg·mL^−1^, IC_50_ (DPPH) = 0.011 mg·mL^−1^, IC_50_ (OH) = 0.261 mg·mL^−1^, IC_50_ (OH) = 0.177 mg·mL^−1^	Liu et al. (2020)
	Pp	*In vitro*	0.04–0.18 mg·mL^−1^			
Activity on skeletal system	EuOCP3	*in vivo*	100, 300 mg·kg^−1^	Male C57BL/6 rats	Ct. B↑, Ct. Th↑, Ct.ar↑, Ct.ar/Tt↑. Chao1↑, Faith’s PD↑, *Shannon*↑*, Allobaculum, Sutterella*↑*, Parabacteroides*↑*,* and *Prevotella*↑. *Dehalobacterium*↓, *Shigella*↓*, Clostridium*↓*, Alistipes*↓. glycerophosphocholine↑, nicotinamide N-oxide↑, glyceric acid↑, cis-9↑, 10-epoxystearic acid↑, oleic acid↑. creatine↓, dioctyl phthalate↓, glycitein↓, linoleic acid↓, 13,14-dihydro-15-keto-PGF2alpha↓, 3-indolepropionic acid↓, glycocholic acid↓, sebacic acid↓, traumatic acid↓. PGK↓, CK↓, ROS↓, JNK↓, GSH-Px↑, CAT↑, SOD↑, ERK↑.	Song et al. (2023)
	EUP (Haochen Biology)	*In vitro*	10, 50, 50, 100, 500 μg mL^−1^	RAW264.7	IL-1β↓., IL-18↓., CD86↓., Wnt10b↑, BMP6↑, TRAP↓, CR↓, MMP9↓, RANKL↓, MCSF↓	Deng et al. (2019)
	EUP-Sr	*In vitro*	10, 50, 50, 100, 500 μg mL^−1^	RAW264.7		
	Ti-EUP-Sr	*In vitro* and *in vivo*	N/A	RAW264.7, HUVECs, HABOBs, HUVECs. Female New Zealand rabbits (2–2.5 kg)	ALP↑, OPN↑, OCN↑, Runx2↑, IL-10↑, VEGF↑, VEGFA↑, EGFL6↑	Sun et al. (2023)
	DPEEK@EUP-Sr	*In vitro*	50, 100 μg mL^−1^	MC3T3-E1	MMP9↓, RUNX2↑, Col1-α1↑	Zhang et al. (2022)
Anti-inflammation activity	Ti-EUP-Sr	*In vitro*	N/A	macrophages	IL-10↑, TNF-α↓, iNOS↓	Sun et al. (2023)
	EUP1	*In vitro* and *in vivo*	10, 25, 50 μg·mL^−1^ 10 mg·kg ^−1^	RAW264.7, male C57BL/6 mice (18-22g)	CD206↑, IL-10↑, TNF-α↓, IL-1β↓, IL-6↓	Li et al. (2017)
	DPEEK@EUP-Sr	*In vitro*	50, 100 μg mL^−1^	MC3T3-E1	IL-1β↓, IL-18↓	Zhang et al. (2022)
	EUP (purity: 60%)	*In vitro* and *in vivo*	50, 100 μg mL^−1^	RAW 264.7, adult male New Zealand white rabbits (2.85 ± 0.2 kg)	IL-6↓, IL-18↓, IL-1β↓, BMP-6↑, Arg-1↑, TGF-β↑	Sun et al. (2021)
	EUP-SeNP	*In vivo*	1.28 mg·mL^−1^	Male C57BL/6JNifdc rats (20–21 g)	DAI↓, CAT↑, GPX↑, T-AOC↑, SOD↑, MDA↓, GSH/GSSH↑, MPO↓, MUC2↑, tight Occludin, Claudin-1↑, Claudin- 3↑, ZO-1↑, IL-1↑, . IL-1β↓, IL-6↓, IL-12↓, IL-17↓, TNF-α↓. *Actinobacteriota*↑, *deferribacterota*↑, *Rikenellaceae*↑, and *Muribaculaceae*↑. campylobacterota↓, colstridia↓, oscillospirales↓, Desulfovibria↓ and Ruminococcaceae↓.	Ye et al. (2023)
Immunomodulatory activity	anti-DEC-205-EUPS-OVA-LPSM	*In vitro* and *in vivo*	5, 10, 20, 40, 80, 160, 320, 640, 1,280 μg·mL^−1^	DCs, female ICR rats (18-22g)	5–1,280 μg·mL^−1^: Increased cell proliferation 160 μg·mL^−1^: Maximum cell proliferation activity Th1↑, Th2↑, IL-4↑ and IFN-γ↑	Feng et al. (2020)
	ELP	*In vitro* and *in vivo*	1,000–10000 μg·mL^−1^ 200 mg·kg ^−1^	RAW 264.7, male BALB/c mice (18-22g)	1,000–5,000 μg·mL^−1^: A570 = 0.436 ± 0.019, 0.599 ± 0.018, 0.606 ± 0.043, 0.674 ± 0.045, 0.717 ± 0.026, respectively. 10,000 μg·mL^−1^: A570 = 0.2284 ± 0.0147 IL-2↑, IL-4↑, IFN-γ↑, TNF-α↑, spleen index = 7.768 ± 0.528, thymus index = 0.645 ± 0.065, WBCs = 4.250 ± 0.339, Peripheral blood lymphocyte density = 2.167 ± 0.291, Phagocytic Index = 4.460 ± 0.079.	Cui et al. (2023)
	EUPS	*In vitro* and *in vivo*	1.2–75 μg·mL^−1^ 0.5 mg	Lymphocyte, DCs, female ICR rats	MHCⅱ↑, CD80↑, CD40↑, CD86↑, IL-4↑, IFN-γ↑, IgG↑, IgG1↑, IgG2a↑, IgG2bl ↑	Feng et al. (2016)
	PsEUL-OVA	*In vitro* and *in vivo*	50, 100, 150, 200, 250, 300, 350 μg·mL^−1^	Macrophage, female ICR rats (18–22 g)	IgG↑, IgG1↑, IgG2a↑, IgG2b↑, IL-2↑, IL-4↑, IL-6↑, IFN-γ↑, CD4+↑, CD8+↑	Feng et al. (2021)
	PsEUL-OVA/Cubs	*In vitro* and *in vivo*	0–350 μg mL^−1^	RAW264.7, DCs, ICR rats (18–22 g)	IL-6↑, IL-4↑, IL-2↑, IgG↑, IgG1↑, IgG2a↑, IgG2b↑, IFN-γ↑, CD4+↑, CD8+↑	Feng et al. (2022)
	EUPS-PLGA	*In vitro* and *in vivo*	1,000, 800, 600, 200, 100, 50, 15, 5 μg mL^−1^	iDC, female ICR mice (18–22 g)	IFN-γ↑, IL-4↑, Th1↑, Th2↑, CD86+↑, CD80+↑, CD40+↑, MHC II↑	Feng et al. (2023)
	anti-DEC-205-EUPS-OVA-PLGA NPs	*In vitro* and *in vivo*	1,000, 800, 600, 200, 100, 50, 15, 5 μg mL^−1^	iDC, female ICR mice (18–22 g)		
	EUPs	*In vivo*	15, 30 mg·kg^−1^	Female BALB/c mice	IgG↓, Anti-ds-DNA↓, anti-ss-DNA↓ and anti-histone anti-bodies↓	Jiang et al. (2011)
Anticomplementary activity	EWDS-1	*In vitro*	23.4–3000 μg mL^−1^	Sheep erythrocytes, Anti-sheep erythrocyte antibody, Anti-sheep erythrocyte antibody, NHS	CH50 = 203 ± 20 μg mL^−1^ AP50 = 45 ± 8 μg mL^−1^	Zhu et al. (2008)
Activity on acute alcoholic injury	EUPL	*In vivo*	10, 20, 40 mg·mL^−1^	Kunming male mice (24–32 g)	ALT↓, AST↓, LPS↓, GSH↑, T-AOC↑, SOD↑, TNF-α↓, IL-6↓, IL-10↓, Occludin↑, Claudin-1↑, ZO-1↑, Proteobacteria↓ and Actinobacteria↓, *Lachnospiraceae*↑ and *Verrucomicrobiaceae*↑,	Li et al. (2024a)
	EUPL	*In vivo*	10, 20, 40 mg·mL^−1^ 5, 10, 20 μg mL^−1^	Kunming male mice (24–32 g)	AChE *e*↑, DA↓, GABA↓, β-EP↓, TNF-α↓, IL-6↓, IL-10↑, GSH↑, SOD↑, NO↑, ROS↓, MDA↓, IL-1β↓, Ca2+↑	Li et al. (2024b)

N/A: means not mentioned.

Osteoarthritis is a degenerative joint disease, classified as a form of aseptic inflammation (Jia et al., 2024a). EUP (purity: 60%) was a type of *E*. *ulmoides* polysaccharide obtained through purchase (Sun et al., 2021). To investigate the inhibitory effect of EUP (purity: 60%) on osteoarthritis and its potential mechanisms, MTT assays were conducted to determine the optimal concentration of EUP (purity: 60%) *in vitro* experiments. Additionally, real-time quantitative polymerase chain reaction (RT-qPCR) was employed to assess the impact of EUP (purity: 60%) on gene expression in RAW 264.7 cells. Concurrently, *in vivo* experiments were performed using a rabbit anterior cruciate ligament transection (ACLT) method to establish an osteoarthritis model. The *in vitro* experimental results indicated that EUP (purity: 60%) at concentrations of 50 and 100 μg mL^−1^ was beneficial for the proliferation of RAW 264.7. The qPCR results demonstrated that EUP (purity: 60%) can inhibit the expression of IL-6, interleukin-18 (IL-18), and IL-1β, and promote the expression of osteogenic and cartilage-related genes bone morphogenetic protein 6 (BMP-6), arginase-1 (Arg-1), and transforming growth factor beta (TGF-β) (Sun et al., 2021). *In vivo* experiments divided the adult male New Zealand white rabbits into three groups: the sham operation group, the OA group, and the EUP group. The results indicated that EUP reduced the extent of cartilage damage, and the degree of trabecular bone separation, while enhancing subchondral trabecular bone density, and increasing the number and thickness of trabecular bones. In addition, EUP (purity: 60%) decreased M1-polarized macrophages while simultaneously increasing M2-polarized macrophages (Sun et al., 2021). Therefore, EUP (purity: 60%) can promote joint cartilage repair and subchondral bone reconstruction, and the regulation of macrophage polarization status may be one of its mechanisms for delaying the progression of osteoarthritis.

**FIGURE 4:**
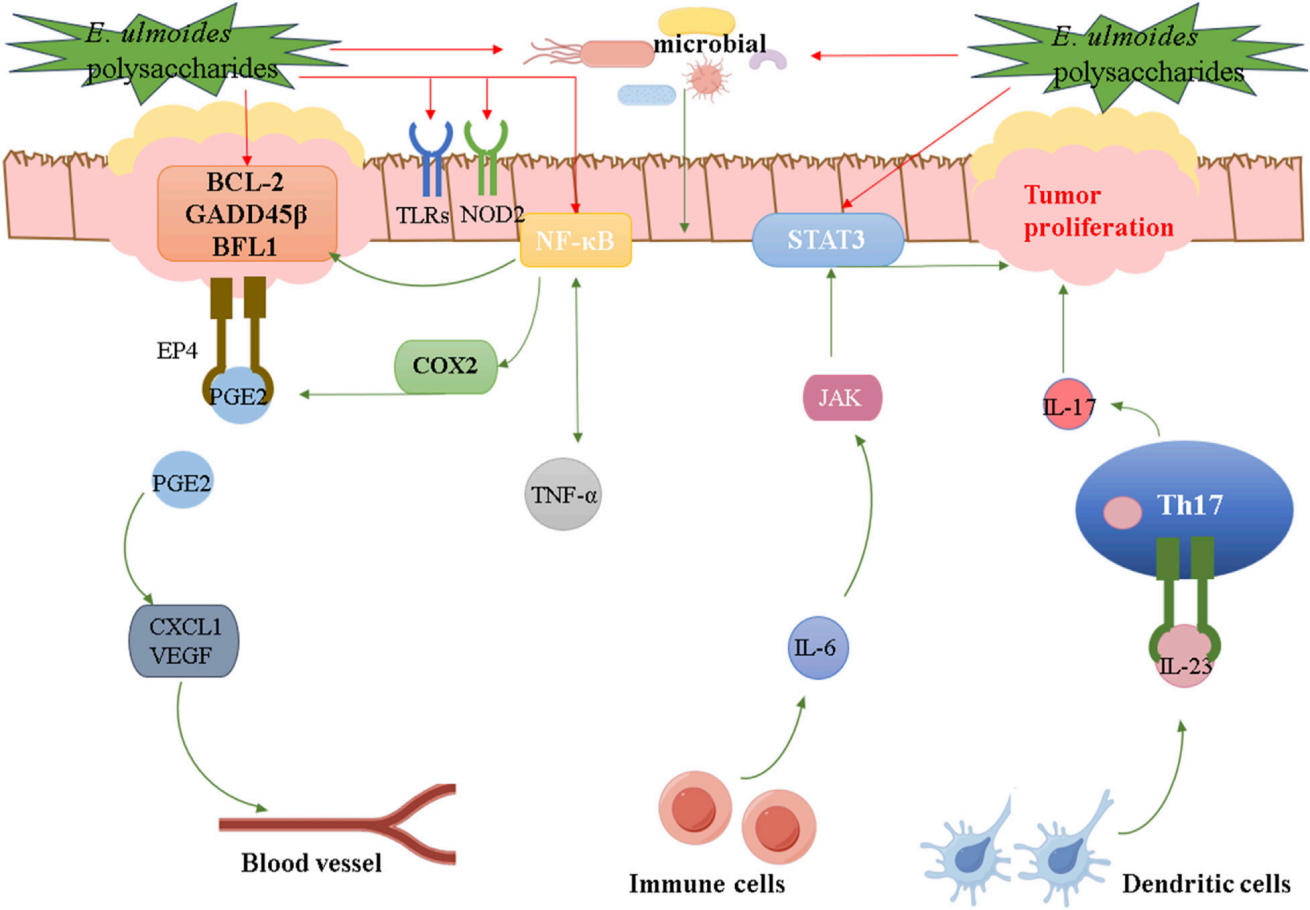
Potential therapeutic pathways for *E*. *ulmoides* polysaccharides on anti-inflammatory activity.

Ulcerative colitis (UC) is a chronic inflammatory disease of the rectum and colon, belonging to aseptic inflammation (Lan et al., 2024). To investigate the effects of EUP-SeNP on 3% dextran sulfate sodium (DSS)-induced UC, C57BL/6JNifdc mice were divided into six groups: control, 3% DSS, 3% DSS + EUP, 3% DSS + SeNP, 3% DSS + EUP-SeNP, and 3% DSS + selenite. The results indicated that EUP-SeNP improved UC by mitigating weight loss, decreasing the disease activity index (DAI), reducing inflammatory cell swelling and intestinal permeability, ameliorating intestinal barrier damage, enhancing the colon’s antioxidant capacity, and modulating the gut microbiota composition. In addition, the mechanism of EUP-SeNP was verified by introducing LPS into intestinal epithelial cell lines, and it was found that EUP-SeNP could attenuate the adverse effects of LPS on cells by enhancing the expression of tight junction proteins, inhibiting the activation of the toll-like receptor 4/nuclear factor kappa-B (TRL-4/NF-κB) pathway, the number of apoptotic cells, and modulating the level of inflammation (Ye et al., 2023). It is worth noting that UC has the potential to transform into colorectal cancer (CRC) (Sasaki et al., 2024). Research has shown that immune signaling pathways such as NF-κB, IL-6/Signal Transducer and Activator of Transcription 3 (STAT3), cyclooxygenase-2/prostaglandin E2 (COX-2/PGE2), interleukin-23/T helper 17 (IL-23/Th17), and TLRs have been confirmed to promote the transition from colitis to CRC, while the role of Nucleotide Binding Oligomerization Domain Containing 2 (NOD2) and gut microbiota also participates in the regulation of inflammatory-cancer transition (Fasano et al., 2024; Khan et al., 2024b). Therefore, in addition to the existing anti-UC mechanisms, there are also some potential therapeutic pathways for *E*. *ulmoides* polysaccharides, as shown in Figure 4. Ti-EUP-Sr and DPEEK@EUP-Sr were also found to have anti-inflammatory activities. In short, *E*. *ulmoides* polysaccharides play different anti-inflammatory role in the treatment of inflammation by various signaling pathways, which is the basis for their applications in the research and development of anti-inflammatory drugs.

### 4.2 Antioxidant activity

Oxidative stress, a negative effect produced by free radicals within the body, is regarded as a significant factor in aging and disease induction (Franco et al., 2024). The identification of antioxidants is precisely targeted at addressing this issue, with natural antioxidants being a focal point. Numerous studies have demonstrated that polysaccharides from *E*. *ulmoides* exhibit significant antioxidant activity.

*In vitro* experiments, the antioxidant capacity of polysaccharides from *E*. *ulmoides* was usually evaluated by measuring the free radical scavenging rate. Many experiments showed that polysaccharides from *E*. *ulmoides* had strong free radical scavenging activity against DPPH,·OH, ABTS and metal ions. These polysaccharides included EULP-MAE, EULP-CHE, EUP-SeNP, crude polysaccharide (Cp) from *E*. *ulmoides* leaves, and its purified polysaccharide (Pp) (Liu et al., 2020; Xu et al., 2018; Ye et al., 2023). *In vivo* experiments, existing research has focused on the protective effects of *E*. *ulmoides* polysaccharide EUP on tissue peroxidation damage and abnormal antioxidant levels during ischemia-reperfusion (IR) - induced nephrotoxicity in male albino rabbits (Hong et al., 2013). The experiment divided the rabbits into a normal group, a renal ischemia-reperfusion (RIR) group, and an EUP treatment group (EUP 300 or 600 mg·kg^−1^). The findings of this study demonstrate that EUP can notably decrease serum Cr and renal IL-6 levels, enhance renal basic fibroblast growth factor (bFGF) levels, significantly reduce renal malonaldehyde (MDA) levels, and elevate glutathione (GSH) levels. Additionally, its dose-dependently reversed the reductions in superoxide dismutase (SOD), catalase (CAT), glutathione peroxidase (GSH-Px), and glutathione reductase (GR) activities in the kidneys of the EUP-treated group to normal levels (Hong et al., 2013). This suggests that EUP exerts its renal protective activity by mitigating IR-induced oxidative damage through its antioxidant and free radical scavenging properties. In summary, polysaccharides from *E*. *ulmoides* exhibit a high antioxidant capacity and boast promising application prospects as functional foods.

### 4.3 Immunomodulatory activity

Immune response is an important physiological process for the recognition and defense against foreign pathogens and toxins (Zhu et al., 2024). Plant polysaccharides are considered as important sources of natural immunomodulators (Yang et al., 2021). The immunomodulatory mechanism of *E*. *ulmoides* polysaccharides is related to its interaction with macrophages, T cells, leukocytes and monocytes.

ELP was a *E*. *ulmoides* leaf polysaccharide obtained via HWE by Cui et al. (2023). In order to investigate the immune-enhancing effect of ELP and evaluate the mechanism, *in vivo* and *in vitro* models were established. *In vitro*, RAW 264.7 cells were treated with ELP at 1,000–10000 μg·mL^−1^ and found that ELP significantly enhanced proliferation and macrophage *in vitro* between 1,000 and 5,000 μg·mL^−1^. *In vivo* experiments, the immunosuppression model of male BALB/c mice was induced with cyclophosphamide (CTX). The mice were divided into three groups: saline (NC), 70 mg·kg^−1^ CTX (MC) or 70 mg·kg^−1^ CTX +200 mg·kg^−1^ ELP (ELP group). The results showed that ELP could protect the immune organs, increase the phagocytic index, and upregulate the expression levels of IL-1β, IL-6, and TNF-α. Furthermore, Western blotting found that ELP improved the levels of phosphorylated p38 Mitogen-Activated Protein Kinase (p38), extracellular regulated protein kinases 1/2 (ERK 1/2) and c-Jun N-terminal kinase (JNK), suggesting that MAPKs may be involved in immunomodulatory effects (Cui et al., 2023). *E*. *ulmoides* bark polysaccharide EUPS was also shown to have immunomodulatory activity through *in vitro* and *in vivo* experiments (Feng et al., 2016). *In vitro* experiments, treatment of Dendritic cells (DC) with EUPS (1.2–75 μg·mL^−1^) increased the expression of major histocompatibility complex (MHC), clusters of differentiation 80 (CD80), clusters of differentiation 40 (CD40) and clusters of differentiation 86 (CD86) on the surface of DCs, indicating that EUPS induced DC maturation. Moreover, EUPS also significantly promoted lymphocyte proliferation and the production of interleukin-4 (IL-4) and interferon gamma (IFN-γ). *In vivo* experiments, EUPS significantly increased specific immunoglobulin G (IgG), immunoglobulin G1 (IgG1), immunoglobulin G2a (IgG2a) and immunoglobulin G2bl (IgG2bl) antibody titers and T cell proliferation in ICR female mice (Feng et al., 2016). These results indicate that EUPS was a strong immunostimulatory agent. Systemic lupus erythematosus (SLE) is a chronic autoimmune disease (Zavaleta-Monestel et al., 2024). EUPs (different from EUPS) was a crude polysaccharide isolated from the stem bark of *E*. *ulmoides* by Jiang et al. through HWE (Jiang et al., 2011). It was found that EUPs could ameliorate CJ-S131-induced SLE in BALB/c mice by inhibiting the increase of serum autoantibodies and total IgG production (Jiang et al., 2011).

In addition to the common *E*. *ulmoides* polysaccharides, some polysaccharide derivatives have also been found to have immunomodulatory activities. To investigate the immunoregulatory activity of PsEUL-OVA, RAW264.7 cells were treated with different concentrations of PsEUL-OVA *in vitro*, and cell viability and phagocytosis were measured. The results showed that PsEUL-OVA (200 μg·mL^−1^) could enhance the proliferation of macrophages and improve their phagocytic efficiency. *In vivo* experiments were conducted using female ICR mice as the experimental subjects, and it was found that PsEUL-OVA significantly increased the titers of IgG, IgG1, IgG2a, and IgG2b and levels of IL-2, IL-4, IL-6, and IFN-γ. Furthermore, it could activate T lymphocytes and promote the maturation of DCs. In summary, PsEUL-OVA induced humoral and cellular immune responses by promoting the phagocytic activity of macrophages and dendritic cells (Feng et al., 2021). It is noteworthy that PsEUL-OVA/Cubs had similar immunoregulatory effects, but its presenting efficiency was higher (Feng et al., 2022). Additionally, anti-DEC-205-EUPS-OVA-LPSM and anti-DEC-205-EUPS-OVA-PLGA NPs had also been proven to have immunoregulatory activity through both *in vitro* and *in vivo* experiments. *In vitro* experiments, anti-DEC-205-EUPS-OVA-LPSM and anti-DEC-205-EUPS-OVA-PLGA NPs could promote the proliferation of dendritic cells and improve their phagocytic efficiency. However, the optimal concentrations were different, with anti-DEC-205-EUPS-OVA-LPSM being 160 μg·mL^−1^ and anti-DEC-205-EUPS-OVA-PLGA NPs being 200 μg·mL^−1^. *In vivo* experiments, both anti-DEC-205-EUPS-OVA-LPSM and anti-DEC-205-EUPS-OVA-PLGA NPs were able to increase IgG antibody levels, promote the cytotoxic activity of cytotoxic T lymphocytes (CTLs) and natural killer cells (NKs) cells, and promote the proliferation of spleen cells (Feng et al., 2020; Feng et al., 2023). Therefore, both of them were able to promote the maturation of dendritic cells and induce both humoral and cellular immune responses.

### 4.4 Anticomplementary activity

The complement system is an important part of the human immune system, mainly including the complement activation pathway, complement lysis effects, and cytotoxic effects (Hamers et al., 2024; Schmidt et al., 2021). When the complement pathway is activated, the complement proteins will gradually form a complex and diverse molecular network, triggering a series of physiological and pathological responses, such as inflammation, tissue damage, autoimmunity, etc. (Lu et al., 2021). Therefore, inhibiting the activation and function of the complement system has become one of the therapeutic strategies for the treatment of many diseases.

EWDS-1 was a uniform protein-bound polysaccharide isolated from the barks of the *E*. *ulmoides* (Zhu et al., 2008). To investigate its anticomplementary activity, it was bio assayed and found that EWDS-1 inhibited complement activation in both classical and alternative pathways, with CH 50 and AP50 values of 203 ± 20 μg·mL^−1^ and 45 ± 8 μg·mL^−1^, respectively. Preliminary mechanistic studies using serum depleted of complement ingredients suggested that EWDS-1 suppressed the activation of the complement system by interacting with C1q, C1r, C1s, C2, C3, C4, C5, and C9. Therefore, EWDS-1 may have a good potential for development in the treatment of complement-related diseases (Zhu et al., 2008). Studies have found that, in addition to the regulation of complement activation and composition, plant polysaccharides can also affect the expression and signaling of complement receptors to achieve anticomplementary activities (Mizuno, 2023). Whether the *E*. *ulmoides* polysaccharides can achieve anticomplement activity through other pathways remains to be investigated.

### 4.5 Activity on acute alcoholic injury

Acute alcoholism refers to an acute condition in which the concentration of alcohol increases sharply and central nervous system inhibition and organ function damage occur due to drinking large amounts of alcohol or drinking highly alcoholic beverages in a short period of time (Szabo and Saha, 2015). Therefore, there is an urgent need to develop natural, safe anti-acute alcoholism drugs. Recent theoretical developments suggest that oxidative stress and inflammation are the key pathophysiological mechanisms of alcoholism (Ghorbani et al., 2016). Studies show that *E*. *ulmoides* polysaccharides can alleviate alcohol-induced liver injury, brain injury and BV-2 microglia dysfunction in mice.

Interactions between the gut microbiota, gut, and liver are critical for the prevention of acute alcoholic liver injury (Esparteiro et al., 2024; Guo et al., 2023). EUPL was a *E*. *ulmoides* leaf polysaccharide that was obtained through HWE by Li et al. (2024b). To study the hepatoprotective effect of EUPL, a model of acute alcoholic liver injury was developed using Kunming male mice. The results showed that EUPL reversed acute alcohol-induced TNF-α, IL-6, IL-10, ALT and AST levels, and significantly increased GSH, SOD and T-AOC levels, thus inhibiting endotoxemia and balancing the homeostasis of the gut-liver axis. In addition, EUPL restored the composition of the intestinal flora by increasing the relative abundance of beneficial bacteria such as *Lactobacillaceae,* and reducing the abundance of harmful bacteria such as *Lachnospiraceae*. It is worth noting that the prolonged pretreatment time of EULP, rather than one injection of EULP, achieved good hepatoprotective effects (Li et al., 2024b). Moreover, EUPL had also been shown to ameliorate alcohol-induced brain injury and BV-2 microglial dysfunction in mice (Li et al., 2024c). An acute alcohol model was developed in Kunming male mice. The results showed that EUPL significantly alleviated neurobehavioral deficits and neurotransmitter damage in mouse brain tissue due by acute alcohol exposure, and also regulated the metabolic disorders in brain tissue. Moreover, EULP improved EtOH-induced reduction in phagocytosis in BV-2 cells, alleviated oxidative stress in microglial BV-2 cells by reducing intracellular ROS and MDA levels, and improved the inflammatory response by reducing the release of NO and inflammatory cytokine IL-1β (Li et al., 2024c). In conclusion, *E*. *ulmoides* polysaccharide EUPL had the role of improving alcoholic liver injury, brain injury and BV-2 microglial dysfunction, which were mainly achieved by regulating oxidative stress and inhibiting inflammatory response. It was found that EULP significantly improved glycerophospholipid metabolism and tryptophan metabolism signaling pathways, in which the three signaling pathways of sphingolipid metabolism, pyruvate metabolism and lysine degradation were also improved to some extent when improving alcoholic brain injury. However, whether there are other metabolic pathways remains to be further studied, and the metabolic pathways that improve alcoholic liver injury are unknown.

### 4.6 Activity on skeletal system

Human and animal skeletal tissues are continually being remodeled, a process that involves the breakdown and resorption of existing bone and the formation of new bone (Serim et al., 2024). OC are responsible for the breakdown and resorption of bone, while osteoblasts (OB) are responsible for new bone formation (Jiménez-Ortega et al., 2024; Liu et al., 2024c). Consequently, OC and OB are pivotal in the metabolic processes of human bones. Existing studies have shown that *E*. *ulmoides* polysaccharides can exert anti-osteoporosis, osseointegration, and bone immunomodulation effects by affecting OC and OB.

**FIGURE 5:**
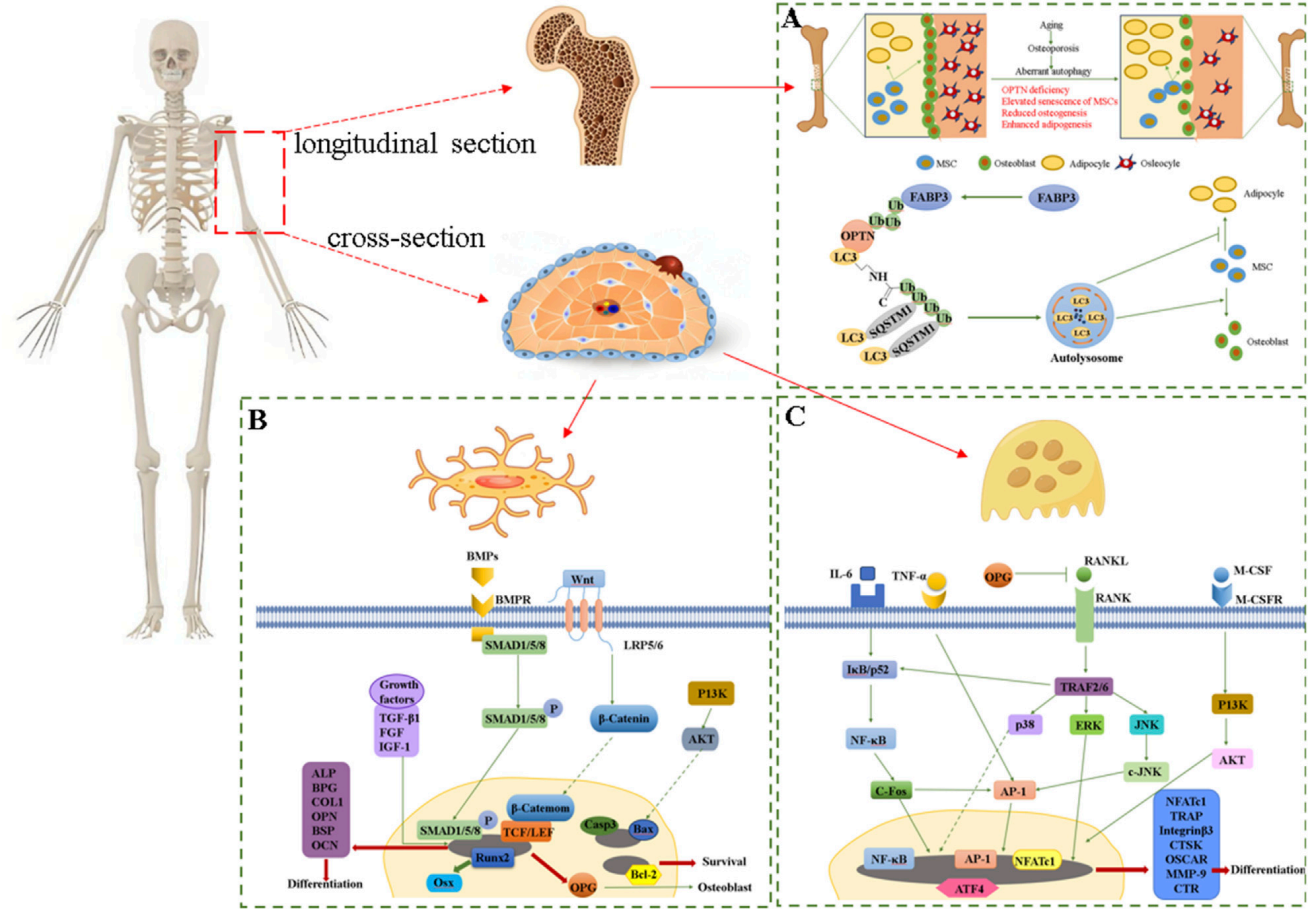
**(A)** Pathogenesis of osteoporosis, **(B)** Signaling factors and pathways that regulate osteoblasts, **(C)** Signaling factors and pathways that regulate osteoclasts.

Osteoporosis (OP) is a prevalent bone disease, characterized by systemic bone loss and microstructural deterioration of bone tissue, the pathogenesis is shown in Figure 5A (Li et al., 2023). In male C57BL/6 rats induced with dexamethasone (Dex) to mimic OP, the polysaccharide EuOCP3 can mitigate reduced bone resorption in the femur and tibia, thinner cortical bone, sparser distal femoral trabeculae, and bone marrow fat accumulation. It also enhanced mineralized bone area, increases osteoblast counts, and decreases osteoclast counts on the cortical bone surface (Song et al., 2023). Furthermore, to explore the *in vivo* mechanism of action of EuOCP3 in OP, additional analyses were performed on the gut microbiota and metabolic profiles. The findings revealed that EuOCP3 bolsters osteogenic function and restores bone metabolism via the extracellular signal-regulated kinase (ERK)/c-Junn-terminal kinase (JNK)/nuclear factor erythroid 2-related factor 2 (Nrf2) signaling pathways. Additionally, *g*_Dorea and *g*_Prevotella exhibited significant alterations post-EuOCP3 treatment, potentially serving as biomarkers for OP in mice treated with EuOCP3 (Song et al., 2023). Notably, the gut microbiota plays a pivotal role in bone metabolism by modulating metabolic processes and the endocrine milieu, thus emerging as a potential therapeutic target for the prevention of OP (Wang et al., 2021).

Bone immunomodulation refers to the promotion of a balanced bone immune microenvironment to accelerate bone regeneration by mediating interactions between immune cells and OB (Mi et al., 2023). The optimal concentration for RAW264.7 cell proliferation was determined to be 50 μg mL^−1^ by CCK8 to detect the cell compatibility of EUP-Sr at different concentrations, and the *in vitro* bone immunomodulation of RAW264.7 to EUP and EUP-Sr was studied at this concentration. The results showed that EUP and EUP-Sr could inhibit the expression of IL-1β, IL-18, B-lymphocyte antigen B7-2 (CD86), macrophage colony-stimulating factor (MCSF), receptor activator of nuclear factor-κB Ligand (RANKL), matrix metallopeptidase 9 (MMP9) and upregulate the expression of recombinant wingless type MMTV integration site family, member 10B (Wnt10b), bone morphogenetic protein 6 (BMP6) (Deng et al., 2019). Therefore, EUP and EUP-Sr should be beneficial to the expression of osteoblast cytokines and affect the expression of osteoclast active genes. Interestingly, EUP-Sr was more effective in reducing the expression levels of IL-1β and MMP9, this is mainly because Sr can inhibit Proinflammatory cytokine IL-1β expression by attenuating activation of the NF-κB pathway and the immune environment created by EUP-Sr can inhibit osteoclast formation (Deng et al., 2019).

Osseointegration denotes a direct and well-defined functional linkage between physiological structures and implants, typically between the implants and the living bone tissue of the human body (Lee et al., 2024). This linkage effectively disperses forces, ensures the stability of the implants, and reduces bone atrophy and resorption (Cao et al., 2024). To study the osseointegration activity of Ti-EUP-Sr, RAW264.7, HUVECs, HABOBs and HUVECs cells were used to perform *in vitro* experiments (Sun et al., 2023). The results showed that the surface functionalization of EUP-Sr could greatly improve the biocompatibility of Ti. Then, the bioactivities of Ti-EUP-Sr to RAW264.7, HABOBs and HUVECs *in vitro* were analyzed by RT-qPCR and enzyme-linked immunosorbent assay. It was found that Ti-EUP-Sr could upregulate the expression of IL-10 in RAW264.7, downregulate the expression of TNF-α and inducible nitric oxide synthase (iNOS) in RAW264.7, and upregulate the expression levels of alkaline phosphatase (ALP), osteopontin (OPN), cyanate and runt-related transcription factor 2 (Runx2) in HABOBs, upregulated Vascular endothelial growth factor A (VEGFA) and epidermal growth factor-like 6 (EGFL6) in HUVECs (Sun et al., 2023). The above results indicated that Ti-EUP-Sr had osseointegration function and promising pro-angiogenic effects, while *in vivo* experiments also demonstrated good osseointegration activity with stable properties. Furthermore, DPEEK@EUP-Sr had also been shown to enhance bone fusion (Zhang et al., 2022). MC3T3-E1 was inoculated to DPEEK@EUP-Sr surface at different concentrations (50, 100, 500 μg mL^−1^) during the study and found that DPEEK @ EUP-Sr promoted the proliferation of MC3T3-E1 cells and 100 μg mL^−1^ was most prominent, while 500 μg mL^−1^ was cytotoxic. The expression levels of osteoblastic cytokine and anti-inflammatory factors were then evaluated by RT-qPCR, which showed that DPEEK@EUP-Sr downregulated IL-1β, IL-18 and MMP9, and upregulated Runx2 and collagen, type I, alpha 1 (Col1-α1), which indicated that DPEEK@EUP-Sr composite had some positive effect on osteogenic and anti-inflammatory effects (Zhang et al., 2022). In short, *E. ulmoides* polysaccharides basically regulate OB and OC by acting on multiple signaling pathways and related factors to achieve the goal of treating the skeletal system. However, the regulatory mechanism of *E. ulmoides* polysaccharides discovered so far is relatively single. Exploring their mechanisms of regulating the skeletal system by regulating other signaling factors of OB and OC may become a new research direction, as shown in Figures 5B, C.

## 5 Structure-activity relationship of *E. ulmoides* polysaccharides

The complex structures of *E*. *ulmoides* polysaccharides and their derivatives have limitations and inconsistency in the understanding of the relationship between their structures and biological activities, which bring challenges to their comprehensive research. It is widely believed that the close connection between the structural characteristics of polysaccharides and their biological activities (Yang et al., 2024). In conclusion, the Mw, monosaccharide composition, the type and position of glycosidic bonds, and the microscopic conformation together influence the biological property of *E*. *ulmoides* polysaccharides.

The Mw of plant polysaccharides is one of the key factors for their biological activities and application values (Yao et al., 2024). In general, the higher the Mw of polysaccharides, the higher their biological activity and application value (Jing et al., 2023). Specifically, the polysaccharide EULP-MAE and EULP-CHE from *E. ulmoides* leaves had Mw of 3.8830 kDa and 1.2055 kDa, respectively, which were found to have antioxidant activities, but EULP-MAE was more active (Xu et al., 2018). In addition, the Mw of EUP was 3.170 kDa and the Mw of EUP-Sr was changed to 14.700 kDa (Deng et al., 2019). However, it was found that EUP and EUP-Sr could downregulate the level of proinflammatory cytokines and reduce the osteoclast active genes to express bone immunomodulation activity. Interestingly, compared with EUP, EUP-Sr tended to reduce the expression level of IL-1β and was more effective in reducing the expression of MMP 9 (Deng et al., 2019).

Monosaccharide composition is one of the key factors affecting the polysaccharide activity, and different monosaccharide composition can significantly affect the anti-inflammatory, antioxidant and other biological activities of polysaccharides (Wang et al., 2024). Therefore, it is of great significance for polysaccharide research and applications to understand and control their monosaccharide composition. For example, both EUPs and EUP1 were polysaccharides obtained from *E*. *ulmoides* bark via HWE, monosaccharide composition of EUPs was Ara, Gal, Glu, Rha, showing immunomodulatory activity, while monosaccharide composition of EUP1 was Ara, Gal, Glu, Rha, Man, showing anti-inflammatory activity (Jiang et al., 2011; Li et al., 2017). This may be attributed to the existence of Man. In addition, the monosaccharide composition of PsEUL was also Ara, Gal, Glu, and Rha, which also showed the role of immunomodulatory, supporting that the difference in biological activities may be attributed to the different monosaccharide composition (Feng et al., 2021). In addition, the proportion of monosaccharide composition can also affect the biological activities. For example, the monosaccharide compositions of EUPL-MAE and EUPL-CHE were Rha, Ara, Gal, Glu, Glu, Xyl, Man, GluA, GalA with ratios 7:4:6:14:1:2:3:1 and 9:3:32:20:55:19:2:14:1, while EUPL-MAE showed stronger antioxidant activity than EUPL-CHE (Xu et al., 2018).

Moreover, structural modifications of polysaccharides can significantly affect the biological properties of polysaccharides (Yang et al., 2022). For example, Sr is a trace element that can promote skeletal growth (Chen et al., 2017). EUP modified with Sr had stronger bone immunomodulatory activity than EUP, while Ti modified on EUP-Sr was conferred anti-inflammatory function and improved osseointegration activity (Deng et al., 2019). In addition, when PEEK was modified on the basis of EUP-Sr, the resulting DPEEK@EUP-Sr was also endowed with significant anti-inflammatory and osteogenic effects, which facilitates the bone fusion of bone and implant (Zhang et al., 2022). structural modification of PsEUL by appropriate methods resulted in PsEUL-OVA, which showed that PsEUL-OVA had a stronger immune enhancement than PsEUL (Feng et al., 2021). Furthermore, EUPS was shown to be immunomodulatory, and nanoparticles targeting the DEC-205 receptor were found to induce an antigen-specific protective immune response. The EUPS was modified with DEC-205 to obtain anti-DEC-205-EUPS-PLGA NPs, and it was found that the immune response of anti-DEC-205-EUPS-PLGA NPs was stronger (Feng et al., 2023).

Structural-activity relationships research is an indispensable link in the process of drug research and development, revealing the drug structure-activity relationship and providing strong support for drug design and optimization. Therefore, extensive scientific research is urgently needed to address this issue.

## 6 Applications and bibliometrics of *E. ulmoides* polysaccharides

### 6.1 Applications

Plant polysaccharides generally have low toxicity and significant biological activities, which makes them have the potential to be developed into pharmaceuticals and functional food supplements (Zou et al., 2024). *E. ulmoides* polysaccharides are commonly used in healthcare products, functional foods and pharmaceuticals due to their many beneficial biological activities. In today’s China market, with “*E. ulmoides* polysaccharide” as keywords in the state food and drug administration website (http://samr.cfda.gov.cn/WS01/CL1029/) for retrieval, received more than 200 by the State Administration for Market Regulation of examination and approval of *E. ulmoides* polysaccharides related health food. Health foods of *E. ulmoides* polysaccharides have the main function such as osteoporosis, immunoregulation. There were 7 dosage forms involved in drug treatment, including pill, capsule, tablet, mixture, cream, wine and granule. Among them, pills are the most common, followed by capsules and tablets. Their pharmacological applications mainly focus on helping to improve alcoholic liver injury, brain injury and promoting osseointegration, while other beneficial activities, such as the treatment of immune diseases through anti-complement activity, still have great potential for development.

So far, there are thousands of patents related to *E*. *ulmoides* polysaccharides worldwide. In product development, *E*. *ulmoides* polysaccharides are natural green, safe, and have excellent biological activities, can be used in the production of *E*. *ulmoides* tea, *E*. *ulmoides* jelly, *E*. *ulmoides* drinks, *E*. *ulmoides* wine and other foods. They can also be used as the main ingredients of anti-osteoporosis, regulate immunity and improve acute alcohol intoxication. Moreover, they are widely used in the field of functional foods. When used as a dietary supplement, *E*. *ulmoides* polysaccharides use their prebiotic activity to regulate the gut flora. At the same time, by adding *E*. *ulmoides* polysaccharides as a feed additive, can promote the growth and development of animals, enhance immunity, promote digestion and absorption and improve product quality (Jansseune et al., 2024). The application of *E*. *ulmoides* polysaccharides, although beneficial, also has many disadvantages. Long-term use of *E*. *ulmoides* polysaccharides can cause digestive system problems, and although it is believed to have a certain liver protection effect, long-term large doses may cause damage to the liver. Moreover, the higher-level structures and conformational relationships of *E*. *ulmoides* polysaccharides have not been elucidated, limiting their application field. It is necessary to further reveal the mechanism of action of *E*. *ulmoides* polysaccharides to promote their wide application in different fields. In short, although the application and development of *E*. *ulmoides* polysaccharides have achieved some results, it is still in the initial stage. Although the functional foods and drugs of *E*. *ulmoides* polysaccharides have been developed to some extent, there are still some problems, such as the small number of products and the single application field. Research on these aspects should be strengthened in future studies to fully realize the application potential of *E*. *ulmoides* polysaccharides.

### 6.2 Bibliometrics

**FIGURE 6:**
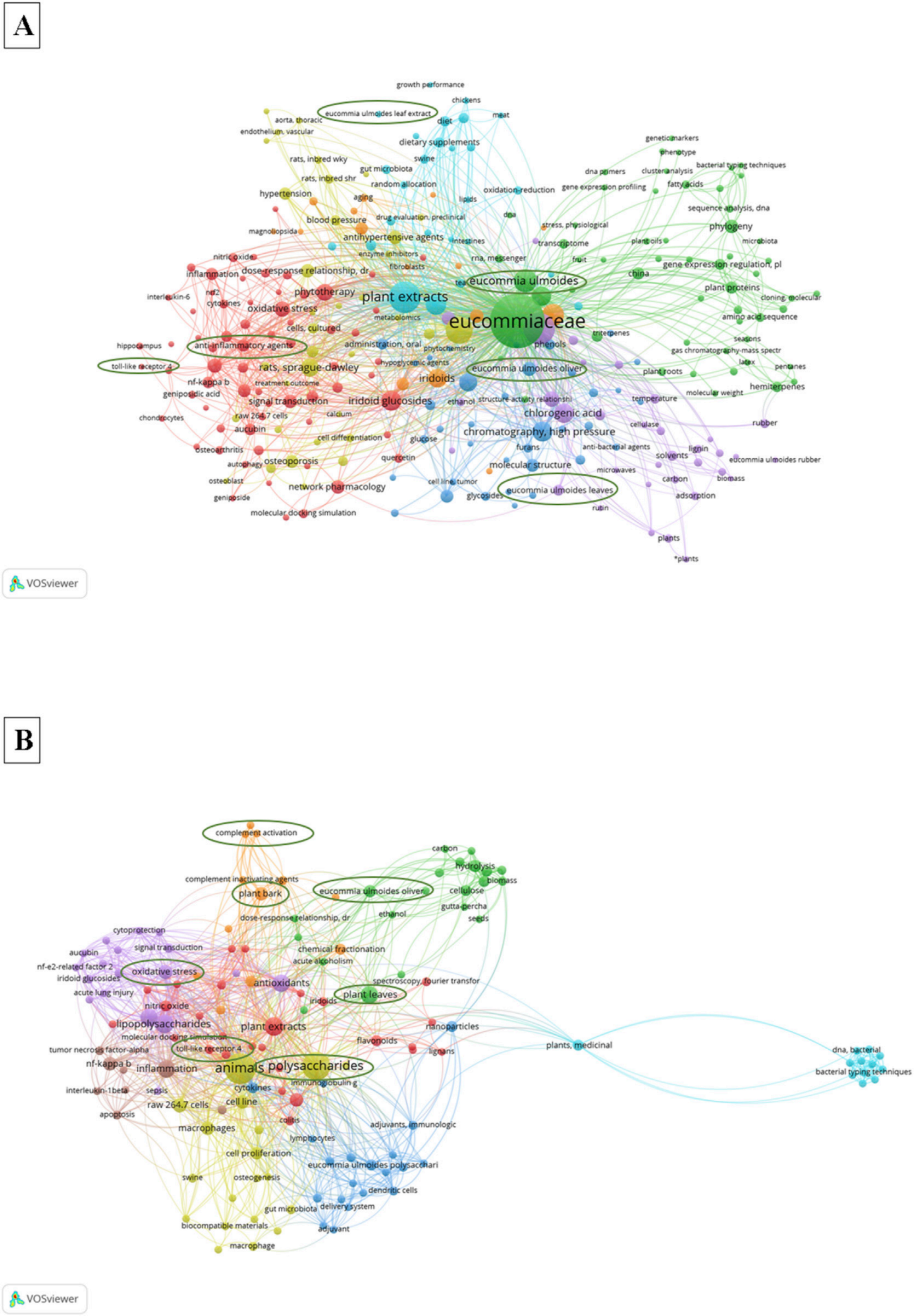
Bibliometrics of *E. ulmoides* and its polysaccharides. **(A)** Bibliometrics of *E. ulmoides*, **(B)** Bibliometrics of *E. ulmoides* polysaccharides.

With the rapid development of digital information and the continuous progress of network technology, bibliometrics also shows important methodological value. Through the bibliometric analysis of the academic research results of *E. ulmoides* polysaccharides, it was found that the current research on *E. ulmoides* polysaccharide mainly focuses on health benefits (such as antioxidant, immune regulation) and signaling pathways, as shown in Figure 6.

## 7 Conclusion and prospectives

**FIGURE 7:**
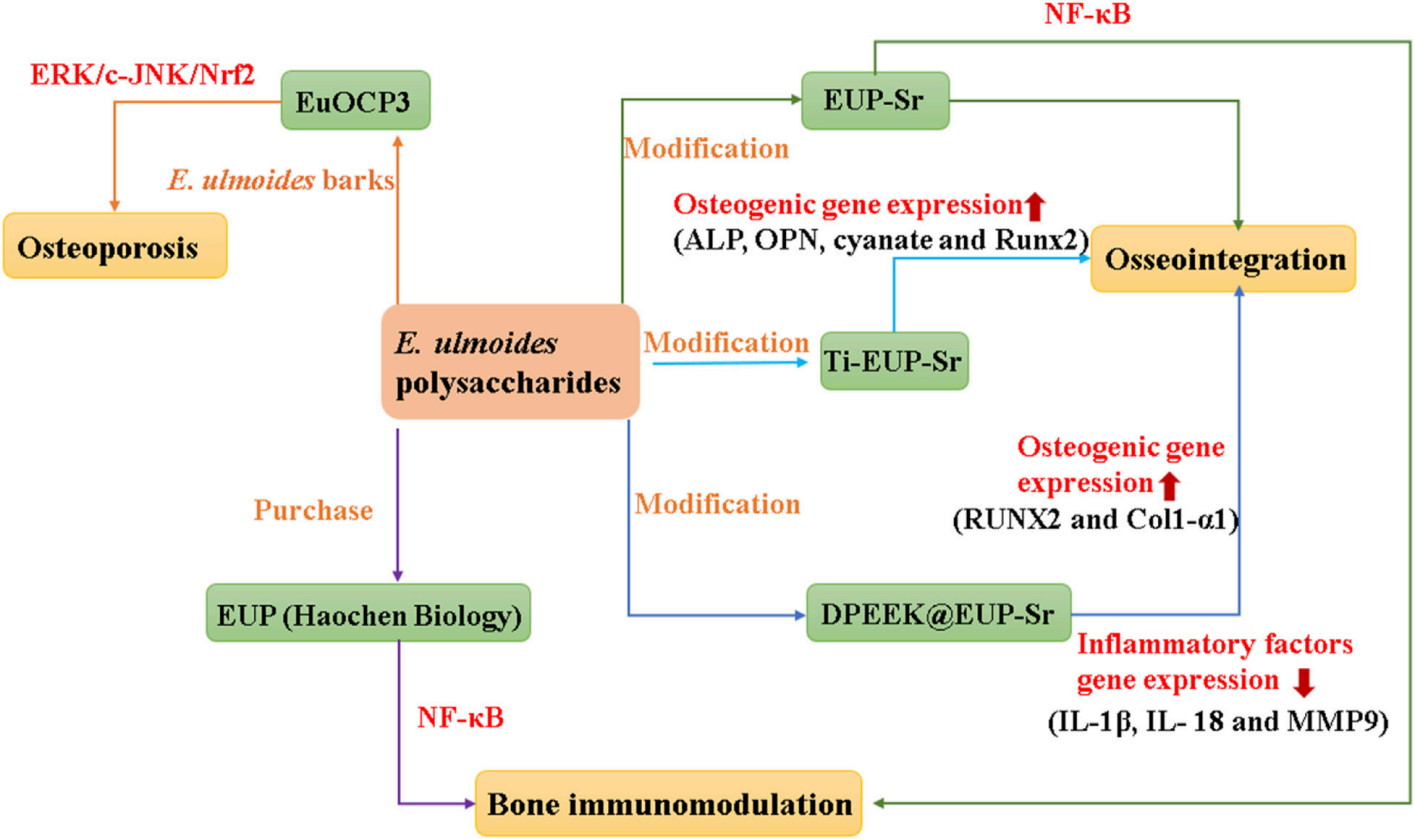
The effect of different *E. ulmoides* polysaccharides on the skeletal system.

*E*. *ulmoides* is a kind of natural plant with edible value and medicinal value, which is commonly used clinically to improve osteoporosis and treat neurodegenerative diseases (Han et al., 2022; Zhang et al., 2024a). In recent years, *E*. *ulmoides* polysaccharides, as one of the main bioactive ingredients of *E*. *ulmoides*, have attracted attention because of their health promotion effect such as anti-osteoporosis, anti-oxidation, anti-inflammation, immune regulation, improvement of acute alcoholism, and promotion of osseointegration. Among them, the effect of *E*. *ulmoides* polysaccharides on the skeletal system is of particular interest, and Figure 7 shows the effect of different *E*. *ulmoides* polysaccharides and their modified polysaccharides on the skeletal system. In addition, because the medicinal *E*. *ulmoides* polysaccharides have no side reaction, are safe and effective, their potential application in the field of functional food has also attracted increasing research. It is now known that factors such as relative Mw, monosaccharide composition, backbone configuration, microscopic conformation and chemical modification influence the activities of *E*. *ulmoides* polysaccharides. However, current studies on the relationship between the structural characteristics and activities of *E*. *ulmoides* polysaccharides are lacking. Further clarification of the advanced structures of *E*. *ulmoides* polysaccharides and their multidimensional correlation with biological activities is needed to elucidate their structural-activity relationships and lay the foundation for the in-depth development of polysaccharide-related products.

Although *E*. *ulmoides* polysaccharides have made progress in many aspects, there are still some obvious problems to be solved. The existing *E*. *ulmoides* polysaccharides extraction methods are mainly HWE and UAE. The HWE is simple to operate but takes a long time. The UAE may shorten the extraction time, but excessive ultrasound frequency may cause polysaccharide loss. Therefore, emerging extraction technologies should be applied to the extraction of *E*. *ulmoides* polysaccharides, such as enzyme-assisted extraction method (EAE) and ultrafiltration. The EAE for the extraction of *E*. *ulmoides* polysaccharides can reduce the extraction conditions, and decompose the *E*. *ulmoides* tissue in relatively mild conditions to accelerate the release or extraction of *E*. *ulmoides* polysaccharides (Riyamol et al., 2023). Ultrafiltration is a new technique to separating the polysaccharides by selective penetration of the membrane (Hidane et al., 2023). Because it is performed at room temperature, it will not destroy the molecular structure of *E*. *ulmoides* polysaccharides. At the same time, because of the small membrane pore size of the membrane concentration system, most of the micromolecular impurities through, play a certain purification role in the *E*. *ulmoides* polysaccharides. The bioactivities of polysaccharides are closely related to their extraction methods and structural features. Therefore, the choice of extraction method is particularly important, and more extraction methods suitable for *E*. *ulmoides* polysaccharides should be explored in the future.

Chemical modification of polysaccharides has become one of the hotspots and directions of new drug development, because it can significantly increase the structural diversity of polysaccharides, enhance the activity, and even produce new biological activities (Tang and Huang, 2024). Nevertheless, there are very few studies on the synthetic biology of *E*. *ulmoides* polysaccharides. Existing studies mainly involve structural modification of *E*. *ulmoides* polysaccharides or preparation of liposome and nanoscale preparations to improve their pharmacological activity and drug delivery capacity. Some studies have shown that the sulfation and deacetylation modification can enhance the immunomodulatory activity of Dendrobium polysaccharides, while the carboxymethylation modification can significantly inhibit it (Deng et al., 2018). Selenized Angelica polysaccharides can significantly promote peripheral lymphocyte proliferation and immune enhancement activity in Newcastle disease vaccine immune chickens (Qin et al., 2013). These may be new ways for *E*. *ulmoides* polysaccharides to enhance the immune response. In addition, the extraction of specific *E*. *ulmoides* polysaccharide synthesis genes and targeted modification to further improve their biological activity is also the focus of future research. By processing the modified *E*. *ulmoides* polysaccharides into liposomes and nanoscale preparations, it can effectively improve their pharmacological activities and drug delivery capacities. It was developed as a drug carrier and delivery system to realize the further analysis and utilization of *E*. *ulmoides* polysaccharides, new drug research and development and safe drug use.

With the increasing attention to *E*. *ulmoides* polysaccharides, *E*. *ulmoides* polysaccharides and their by-products came on the market, and the standard questions about their yields and dosages also appeared. At present, the standard used by the Chinese Pharmacopoeia is usually the thickness or the filament content, but this standard is not very standard. Using micromolecular ingredients such as lignin as potential indicators of harvest time also has the problem of being easily degraded. Therefore, the polysaccharides with high content and stable properties in *E. ulmoides* can be used as an important indicator for judging the quality of *E. ulmoides* and its products. In addition, gutta-percha, as an isomer of natural rubber, is an important part of the *E. ulmoides* industry chain and is usually derived from *E. ulmoides* barks (Zhang et al., 2008). However, without the protection of the bark, the *E. ulmoides* tree is difficult to survive, making it a disposable product and causing a waste of resources. Modern research has found that gutta-percha is also present in *E. ulmoides* leaves. Using *E. ulmoides* leaves to extract gutta-percha not only makes sustainable use of resources, but the leaves after extraction can also be used to obtain *E. ulmoides* polysaccharides to achieve the comprehensive development and utilization of *E. ulmoides*, which is an effective way to promote the industrialization of *E. ulmoides*.

This review provides an important reference for further understanding of the extraction, purification, modification, structural characteristics, microscopic conformation, and biological activities of *E*. *ulmoides* polysaccharides. It lays a foundation for its potential applications in food, healthcare products, medicine and other fields. At the same time, it summarizes the basic research of *E*. *ulmoides* polysaccharides and points out the problems existing in the contemporary development, which provides guidance for the development and utilization of other polysaccharides.
